# METTL1/WDR4 Complex: A Novel Epitranscriptomic Target at the Crossroads of RNA Methylation and Drug Discovery

**DOI:** 10.1021/acs.jmedchem.6c01379

**Published:** 2026-07-20

**Authors:** Emanuele Fabbrizi, Gebremedhin Solomon Hailu, Andrea Mancini, Antonello Mai, Francesco Fiorentino, Dante Rotili

**Affiliations:** † Department of Drug Chemistry and Technologies, 9311 Sapienza University of Rome, 00185 Rome, Italy; ‡ Department of Science, 128165 Roma Tre University, 00146 Rome, Italy; § Department of Biochemical Sciences, Sapienza University of Rome, 00185 Rome, Italy; ∥ Biostructures and Biosystems National Institute (INBB), 00165 Rome, Italy

**Keywords:** METTL1/WDR4, RNA methylation, methyltransferases, cancer therapeutics, epitranscriptomics

## Abstract

METTL1, in complex with its partner protein WDR4, is the principal writer of internal and RNA-associated N^7^-methylguanosine (m^7^G), an epitranscriptomic modification that remodels translation, RNA stability, and stress-response pathways. Across diverse cancer types, including hepatocellular carcinoma, cholangiocarcinoma, lung and bladder cancer, glioma, AML, and prostate cancer, METTL1/WDR4-driven expansion of m^7^G-modified tRNAs and stabilization of codon-biased mRNAs amplifies oncogenic programs governing cell-cycle progression, EMT, DNA repair, and immune evasion. Context-specific roles extend to autoimmune, cardiovascular, and neurological disorders, where METTL1 regulates hypertrophy, fibrosis, angiogenesis, neurodevelopment, and inflammation. Recent advances have established METTL1 as a tractable methyltransferase target: fragment-derived SAM-pocket ligands provide optimal starting points, and recently disclosed THIQ-based inhibitors report nanomolar inhibition, robust cellular target engagement, and functional suppression of tRNA m^7^G in cells. These advances provide the first chemical foothold for therapeutic modulation of m^7^G pathways and underscore METTL1 as a promising yet complex target requiring careful biological stratification.

## Graphical abstract

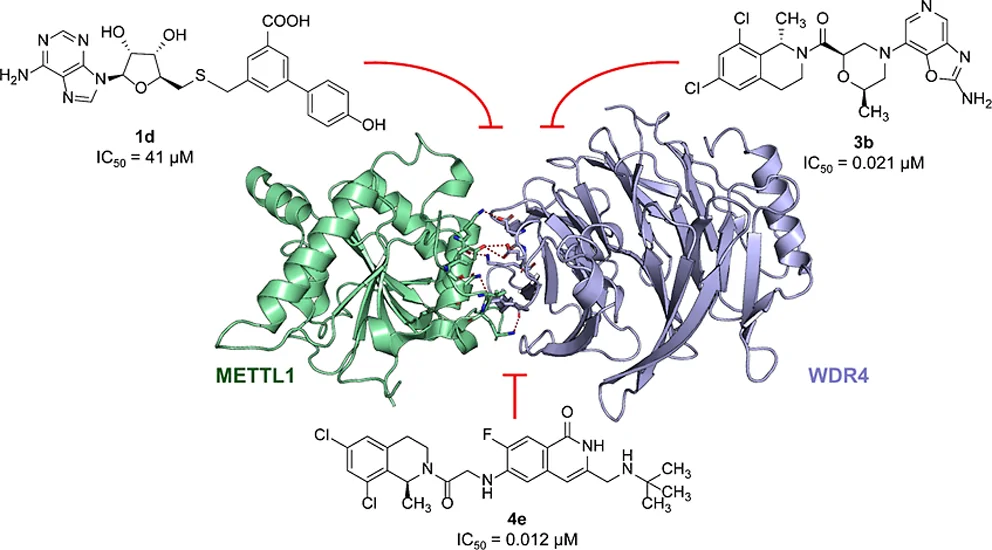

## Significance, Impact, and Innovation

This Perspective establishes the METTL1/WDR4 complex as an emerging epitranscriptomic target linking m^7^G RNA methylation to cancer and other human diseases. It provides a comprehensive framework integrating molecular mechanisms, structural biology, disease relevance, and medicinal chemistry. By highlighting the first potent METTL1 inhibitors together with structure-guided opportunities for selective drug design, it defines the current state of the field and outlines future directions for developing innovative epitranscriptomic therapeutics.

## Introduction

RNA methylation has become recognized as a central regulatory layer in eukaryotic gene expression, influencing nearly every dimension of RNA metabolism, from splicing and nuclear export to transcript stability, translation efficiency, and controlled decay.^1,2^ Within this chemically diverse landscape, methyltransferase-like (METTL) proteins constitute one of the two major methyltransferase superfamilies in mammalian cells, the other being SET-domain lysine methyltransferases.^3^ METTL enzymes share a highly conserved Rossmann-like seven−β-strand (7BS) fold that binds the cosubstrate *S*-adenosyl-l-methionine (SAM) and catalyzes methyl transfer to nucleic acids or, in certain cases, proteins.^4^ More than thirty-four METTL paralogues have been identified in humans, and although research has historically emphasized the m^6^A methyltransferase complex composed of METTL3 and METTL14, it is increasingly evident that several other METTL enzymes mediate functionally distinct methylation reactions with major implications for cell physiology and disease.^5^ Among these, METTL1 and its obligate binding partner WDR4 have attracted particular attention as primary writers of m^7^G in tRNA, selected mRNAs, and pri-miRNAs.^1,6,7^ From a medicinal chemistry perspective, METTL1 is particularly attractive because it is a class I SAM-dependent enzyme with a well-defined cosubstrate pocket, measurable *S*-adenosyl-l-homocysteine (SAH) forming activity in biochemical assays, and multiple orthogonal cellular pharmacodynamic readouts. These include direct target engagement assays and quantification of the hallmark tRNA m^7^G46 mark, enabling clear linkage between binding, pathway modulation, and phenotype. At the same time, the obligate partnership of METTL1 with WDR4 creates composite surfaces, at the heterodimer interface and along the RNA-binding channel, that might be exploited for non-SAM-competitive or allosteric inhibition strategies.^8^

### The m^7^G Modification

m^7^G is one of the most evolutionarily ancient RNA modifications and appears in multiple biochemical contexts.^9^ The most familiar form is the 5′ cap structure that marks RNA polymerase II transcripts, where N^7^-methylation of guanosine by RNMT (RNA Guanine-7 Methyltransferase), aided by its cofactor RNMT-Activating Miniprotein (RAM), promotes efficient splicing, engages the nuclear cap-binding complex, facilitates 3′-end processing and export, and primes mRNAs for cytoplasmic translation initiation through eIF4E.^10^ Beyond its canonical role in translation, the cap influences miRNA maturation and participates in pathways of RNA quality control. Yet, m^7^G is not restricted to the 5′ *terminus*. Internal m^7^G residues occur in mRNAs, rRNAs and, more prominently, in tRNAs. These internal modifications exert structural effects that modulate RNA folding landscapes, relax local secondary structure, and enhance ribosomal access.^6,11^ In mRNAs, internal m^7^G appears enriched in AG-rich motifs, particularly within 5′ untranslated regions (UTR), where it can unwind inhibitory structures and improve initiation efficiency.^6,11^ Although some mapping approaches initially suggested widespread internal m^7^G across the transcriptome, more chemically stringent methodologies have challenged this view. As a result, the true prevalence of internal m^7^G in mRNA remains a matter of ongoing investigation, with growing consensus that the mark is selectively installed on a subset of biologically relevant transcripts rather than being globally distributed.^6^

The most conserved site of internal m^7^G modification lies at position 46 in the variable loop of tRNA.^9^ This modification has been preserved from yeast to mammals and plays a decisive role in tRNA folding, stabilization of the tertiary core, and maintenance of decoding fidelity.^12,13^ Methylation at G46 prevents the accumulation of misfolded tRNA species, protects tRNAs from endonucleolytic cleavage, and reduces ribosomal pausing during elongation, particularly at codons decoded by m^7^G-modified tRNAs.^1^ Among these, the arginine isoacceptor Arg-TCT-4–1, which decodes AGA codons, is especially critical. Efficient translation of AGA-enriched transcripts depends heavily on the availability of m^7^G-modified Arg-tRNA, and these transcripts frequently encode cell-cycle regulators, metabolic enzymes, growth factor receptors, and stress-response proteins. Thus, the METTL1/WDR4 complex imposes a codon-biased translational program, shaping the proteome in ways that directly impact proliferation, differentiation, and cellular adaptation to stress.^1,14,15^

The influence of m^7^G extends to the microRNA pathway as well. Many primary miRNAs (pri-miRNAs) contain G-quadruplex structures that hinder Drosha processing.^16^ m^7^G deposition can destabilize these inhibitory conformations, enabling efficient cleavage and increasing mature miRNA abundance. The let-7 family represents a well-characterized example: METTL1-dependent m^7^G accelerates pri-let-7 processing and strengthens its tumor-suppressive repression of HMGA2, RAS, and MYC.^11,17^ This dual signaling effect, in which METTL1 supports oncogenic growth through tRNA methylation while potentially acting as tumor suppressor through enhanced miRNA maturation, highlights the complex functional logic of m^7^G modification and underscores the importance of the cellular context.

Three enzymatic systems mediate m^7^G deposition in mammalian cells, each possessing distinct substrate specificity and regulatory architecture. RNMT catalyzes cap m^7^G installation in a process intimately coupled to transcription initiation. WBSCR22 (Williams-Beuren syndrome chromosome region 22), in complex with TRMT112 (tRNA methyltransferase activator subunit 11–2), methylates G1639 of 18S rRNA and is essential not so much for the methylation event itself as for its structural role in pre-40S ribosomal subunit export.^18^ Dysregulation of WBSCR22 is common in malignancy and contributes to aberrant ribosome biogenesis and survival signaling.^19^ The third system, based on METTL1 and WDR4, is responsible for internal m^7^G in tRNA, pri-miRNA, and selected mRNA sites and is emerging as a major determinant of translational reprogramming in cancer and development.

### METTL1/WDR4: Overview

METTL1 is encoded on chromosome 12q13–14 and belongs to the class I of SAM-dependent methyltransferases.^20^ Its expression profile in healthy human tissues is uneven, with high levels in the pancreas, kidney, bladder, and reproductive tissues, low expression in the central nervous system, and minimal detection in ocular tissues. In contrast, many tumors, including hepatocellular carcinoma (HCC), intrahepatic cholangiocarcinoma (ICC), lung adenocarcinoma, bladder cancer (BCa), esophageal squamous cell carcinoma (ESCC), nasopharyngeal carcinoma (NPC), and gastric cancer (GC), display marked METTL1 overexpression. Elevated METTL1 correlates with tumor aggressiveness, metastatic behavior, and reduced survival, suggesting that aberrant enhancement of m^7^G-dependent translation provides a proliferative advantage.^6^

METTL1 relies on WDR4, a seven-bladed β-propeller protein, for stability, active site organization, and substrate recognition. Loss of WDR4 leads to decreased METTL1 abundance, almost complete loss of m^7^G46 in tRNA, and severe translational defects.^21^ In humans, biallelic WDR4 mutations cause microcephalic primordial dwarfism, underscoring the importance of METTL1/WDR4-dependent translational control during neurodevelopment. Mechanistically, disruption of the METTL1/WDR4 complex impairs the function and abundance of m^7^G-modified tRNAs, leading to ribosome pausing at codons normally decoded by these tRNAs, reduced translation of codon-enriched developmental transcripts, and broader disturbances in proteostasis.^21^

Regulation of METTL1 integrates growth-factor signaling, transcriptional control, and chromatin state. One major regulatory mechanism is phosphorylation of METTL1 by Protein Kinase B (AKT) and Ribosomal S6 Kinase (RSK), which modulates its activity and stability in response to extracellular cues.^22^ Oncogenic transcription factors such as c-MYC can further enhance expression of METTL1 or WDR4, while epigenetic remodeling, such as p300/SP1-driven H3K27 acetylation at the METTL1 promoter, amplifies transcription in certain malignancies.^15,23^ These multilayered inputs create a signaling axis in which mitogenic pathways and chromatin regulators converge on METTL1 to recalibrate the translational landscape.

The biological consequences of METTL1-mediated m^7^G installation in tRNA are profound. Methylation at G46 reinforces key intramolecular interactions that stabilize the tRNA tertiary fold, thereby reducing misfolding and fragmentation. Functionally, this stabilization supports ribosomal elongation, preventing pausing and collision at codons decoded by m^7^G-modified tRNAs.^22^ Many mRNAs enriched for such codons encode proteins required for cell-cycle progression (e.g., cyclins A2, D2, D3, and E1, and CDK6/8) and growth factors like epidermal growth factor receptor (EGFR) and vascular endothelial growth factor A (VEGFA). In cancer cells, loss of METTL1 reduces translation of these proliferative drivers, leading to impaired growth and invasion. Conversely, METTL1 overexpression enhances codon-biased translation and supports oncogenic phenotypes.^6,15^

Although the m^7^G cap is installed exclusively by RNMT, METTL1/WDR4 also appears capable of depositing internal m^7^G marks on mRNAs.^15^ These internal sites can modulate translation by relaxing local secondary structure and thereby improving initiation efficiency.^15^ However, the prevalence of this modification remains debated. Antibody-based mapping has suggested widespread internal m^7^G, whereas chemical sequencing and mutation-assisted profiling often fail to detect a global pattern, raising concerns about antibody cross-reactivity and overestimation of modification density. Despite this technical uncertainty, functional evidence from specific cancers, such as HCC and nasopharyngeal carcinoma, indicates that METTL1-mediated internal m^7^G can meaningfully influence translation of selected transcripts.

METTL1/WDR4 also plays an important role in miRNA biogenesis. The installation of m^7^G within G-rich motifs of pri-miRNAs disrupts G-quadruplex structures, which impede Drosha cleavage. This conformational remodeling accelerates production of pre-miRNA and ultimately increases levels of mature miRNAs. The let-7 family exemplifies this mechanism: enhanced m^7^G-dependent processing increases let-7 levels and reinforces repression of oncogenic targets. Interestingly, the net effect of METTL1 on tumor progression may depend on whether its tRNA-centered or miRNA-centered activities dominate in a given cellular context. While increased m^7^G-tRNA generally promotes oncogenesis via enhanced translation of proliferative genes, enhanced let-7 maturation may suppress tumorigenesis, illustrating the context specificity of METTL1 function.^15^

### Structural Features of the METTL1/WDR4 Complex

The human METTL1/WDR4 complex constitutes a paradigmatic example of a class I SAM–dependent RNA methyltransferase whose catalytic competence is intrinsically coupled to assembly with an auxiliary β-propeller partner, and recent crystallographic (Figure 1A) and cryo-electron microscopy (cryo-EM) studies (Figure 1B) have elucidated in detail how this heterodimeric architecture underpins catalysis, regulation, and tRNA substrate recognition.^6,7^ METTL1 adopts a canonical Rossmann-like fold characteristic of class I methyltransferases, organized around a central seven-stranded β-sheet flanked by α-helices that shape the SAM-binding pocket and the substrate channel, but is functionally specialized for m^7^G installation at position G46 of tRNA.^6^ The core catalytic domain, spanning approximately residues 77–259, comprises the conserved SAM-dependent methyltransferase scaffold, while the *N*-terminal segment (residues 1–33) remains intrinsically flexible in crystal structures yet is evolutionarily conserved and emerges as a critical regulatory element. WDR4 folds into a seven-bladed WD-repeat β-propeller arranged in a toroidal architecture, with a C-terminal α-helix that folds back between blades 1 and 7, contributing to an extensive and highly structured interface with METTL1 (Figure 1A).^6,7,24^ The heterodimer buries roughly 1000–1700 Å^2^ of surface area, depending on the construct analyzed, through a dense network of hydrophobic contacts, salt bridges, and hydrogen bonds, rendering METTL1 catalytically competent. Key interfacial interactions include polar contacts between WDR4 D166/E167 and METTL1 K143, as well as interactions involving METTL1 Y37, K40, and N147 with residues on WDR4 blades 3 and 4; disruption of these contacts by alanine substitution or charge reversal destabilizes the complex and abolishes methyltransferase activity in vitro and in cellular rescue assays, underscoring that heterodimerization is indispensable for function. Disease-associated mutations further emphasize this principle: WDR4 R170, located at the junction of blades 3 and 4 and involved in stabilizing the β-propeller core rather than directly contacting METTL1, is mutated in MPD, and R170L or R170Q variants markedly impair enzymatic activity by compromising structural integrity of the scaffold.^6,24^ At the catalytic center, SAM binding within METTL1 is mediated by a constellation of conserved residues that precisely orient the cosubstrate for the methyl transfer (Figure 1A). Structural analyses reveal that the adenine ring of SAM occupies a hydrophobic pocket defined by I108, A141, and M142 and is anchored by hydrogen bonds involving N140 and backbone atoms, while the ribose hydroxyl groups interact with E107, and the methionine carboxylate is coordinated by T238 and the backbone NH of E240.^7,24^ Mutagenesis of these residues uniformly disrupts methylation, validating their functional importance. Importantly, WDR4 binding induces subtle, but functionally consequential conformational rearrangements within METTL1: the loop encompassing residues 107–112 intrudes toward the SAM pocket, with I108 and R109 shifting inward, while additional plasticity is observed in the β3−α3 loop and helix α6, and the β4−α4 loop (residues 161–175) adopts distinct conformations depending on the assembly state. These changes rationalize the marked enhancement of SAM affinity upon heterodimer formation, as isothermal titration calorimetry demonstrates a reduction in the dissociation constant from ∼20 μM for METTL1 alone to ∼4 μM for the METTL1/WDR4 complex, explaining why the isolated catalytic subunit exhibits weak or inconsistent activity. Beyond cosubstrate binding, METTL1 and WDR4 cooperate extensively in tRNA recognition.^7^ Electrostatic surface mapping reveals a continuous basic channel spanning the METTL1 active site and WDR4 blades 3 and 4, ideally suited to engage the phosphate backbone of the tRNA T-arm and variable loop. WDR4 contributes directly to substrate docking through clusters of lysine and arginine residues, including R103, R104, K83, R165, and R170, whose mutation selectively impairs tRNA binding and the catalytic activity without altering SAM affinity. Electrophoretic mobility shift assays (EMSA) corroborate this division of labor, showing that METTL1 alone binds tRNA weakly and heterogeneously, whereas the heterodimer forms stable, high-affinity complexes in the nanomolar range.^7,24^ On the METTL1 side, substrate engagement is mediated by inducible structural elements, most notably a transient 3_10_ helix (αC) formed by residues 164–173 and helix α6, which reposition upon tRNA binding to insert into the tRNA elbow and stabilize the G46-containing region. Cryo-EM analyses reveal that this induced-fit mechanism is coupled to bending of the tRNA anticodon arm, positioning the variable loop and target guanosine optimally for base flipping into the catalytic pocket (Figure 1B).^6^ The flexible N-terminal region of METTL1 further integrates regulatory inputs with catalysis. Residues 16–33 transiently associate with the catalytic core near the active site entrance, with H26 and S27 occupying a strategic position that couples N-terminal dynamics to SAM binding and tRNA engagement. Truncation beyond residue 20 or mutation of K18 or H26 abolishes activity, highlighting the functional importance of this regulatory “plug.” Notably, S27 is an AKT phosphorylation site, and structural modeling combined with NMR data indicates that phosphorylation or phosphomimetic substitutions introduce steric and electrostatic clashes that destabilize the *N*-terminal core interaction and interfere with tRNA docking, providing a direct structural mechanism for signal-dependent regulation of METTL1 activity.^6^ At the chemical level, modeling of G46 within the active site supports a SN_2_ methyl transfer mechanism in which precise geometric alignment, rather than classical general acid–base catalysis, governs reactivity, with residues such as R109, D163, E240, and H26 contributing to substrate positioning and transition-state stabilization.^6^

**Figure 1:**
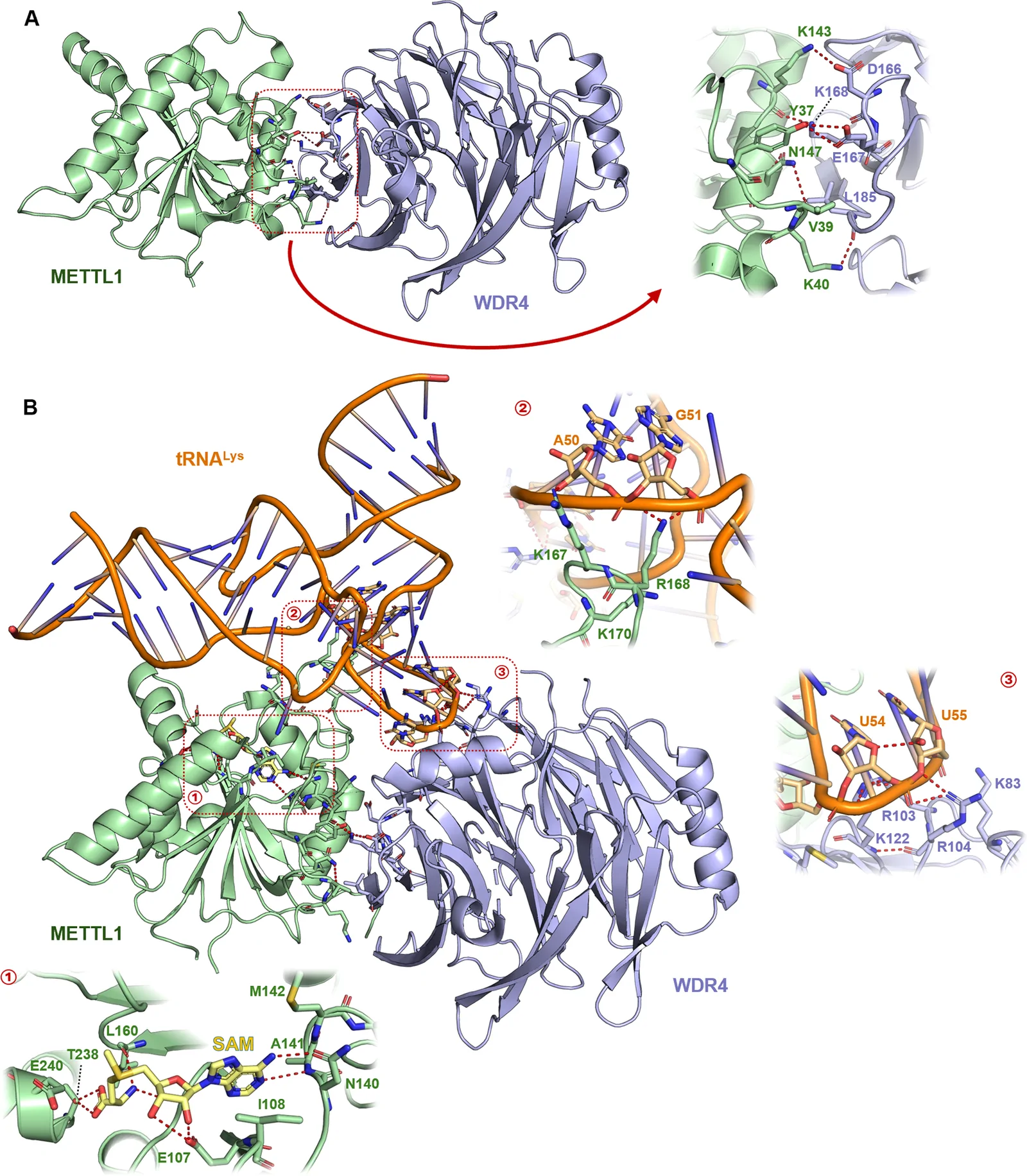
(A) Crystal structure of the METTL1/WDR4 complex (PDB ID: 7U20). Inset: close-up view of the METTL1/WDR4 interface highlighting key interfacial interactions. METTL1 is shown in green and WDR4 in light blue. Key residues are shown as sticks. Hydrogen bonds are depicted as red dotted lines. (B) Cryo-EM structure of the METTL1/WDR4 complex bound to SAM and the tRNA^Lys^ substrate. Inset 1: close-up view of key interactions between METTL1 and SAM. Inset 2: close-up view of key interactions between METTL1 and tRNA^Lys^. Inset 3: close-up view of key interactions between WDR4 and tRNA^Lys^. METTL1 is shown in green, WDR4 in light blue, and tRNA^Lys^ in orange; SAM is shown as yellow sticks. Key residues are shown as sticks. Hydrogen bonds are depicted as red dotted lines.

From a drug-discovery standpoint, the structures highlight three actionable opportunities: (i) the canonical SAM pocket, where anchoring interactions repeatedly involve residues in the adenine subsite and the D163/R109 region; (ii) a peripheral rim around the donor site shaped by flexible elements (including the 107–112 loop and the β4−α4 region) that undergo WDR4-dependent rearrangements and may support induced-fit or allosteric inhibition; and (iii) the METTL1/WDR4 interface and composite RNA-binding groove, which provide heterodimer-specific surfaces absent in monomeric methyltransferases and may offer selectivity advantages. These architectural features argue that METTL1 can be targeted not only via SAM competition, but also via interface- or conformation-selective chemotypes.^6,7^

A central challenge in METTL1 inhibitor development is achieving selectivity within the highly conserved landscape of human class I SAM-dependent methyltransferases. The Rossmann-like fold responsible for SAM recognition is structurally conserved among several methyltransferases, including RNMT, GNMT, and the METTL3/METTL14 complex, creating potential liabilities for adenine-like or SAM-competitive small molecules. Therefore, future medicinal chemistry efforts should avoid relying exclusively on interactions within the conserved adenine-binding region and instead exploit METTL1-specific structural determinants.

Several strategies may improve selectivity. First, extension of SAM-competitive inhibitors toward less conserved peripheral regions surrounding the cosubstrate pocket could enable recognition of METTL1-specific residues and induced-fit conformations. Second, the composite RNA-binding channel generated by the METTL1/WDR4 heterodimer represents an attractive opportunity, as residues involved in substrate positioning and tRNA recognition are less conserved across methyltransferase families. Finally, targeting the METTL1/WDR4 protein–protein interface may provide the highest level of selectivity because this surface is unique to the functional heterodimer and absent in monomeric methyltransferases. Although disrupting large protein–protein interfaces remains challenging, fragment-based approaches, molecular glues, or compounds stabilizing inactive conformational states may represent feasible future strategies. Overall, the next generation of METTL1 inhibitors will likely require a transition from purely SAM-mimetic molecules toward structure-guided chemotypes combining catalytic-pocket anchoring with interactions involving METTL1/WDR4-specific structural features. m^7^G RNA Methylation by METTL1/WDR4 in Cardiovascular, Neurological, Autoimmune, and Other Diseases.

Accumulating evidence indicates that METTL1 is involved in tumorigenesis and plays a critical role in the initiation, progression, and prognosis of a broad spectrum of nonmalignant diseases. Aberrant expression or dysfunction of METTL1 has been reported in multiple pathological conditions, where it contributes to disease progression primarily through dysregulation of immune responses, cellular differentiation, protein synthesis, and metabolic homeostasis. In this section, we summarize the current understanding of METTL1 function in nontumor diseases and discuss its potential therapeutic relevance (Figure 2).

**Figure 2:**
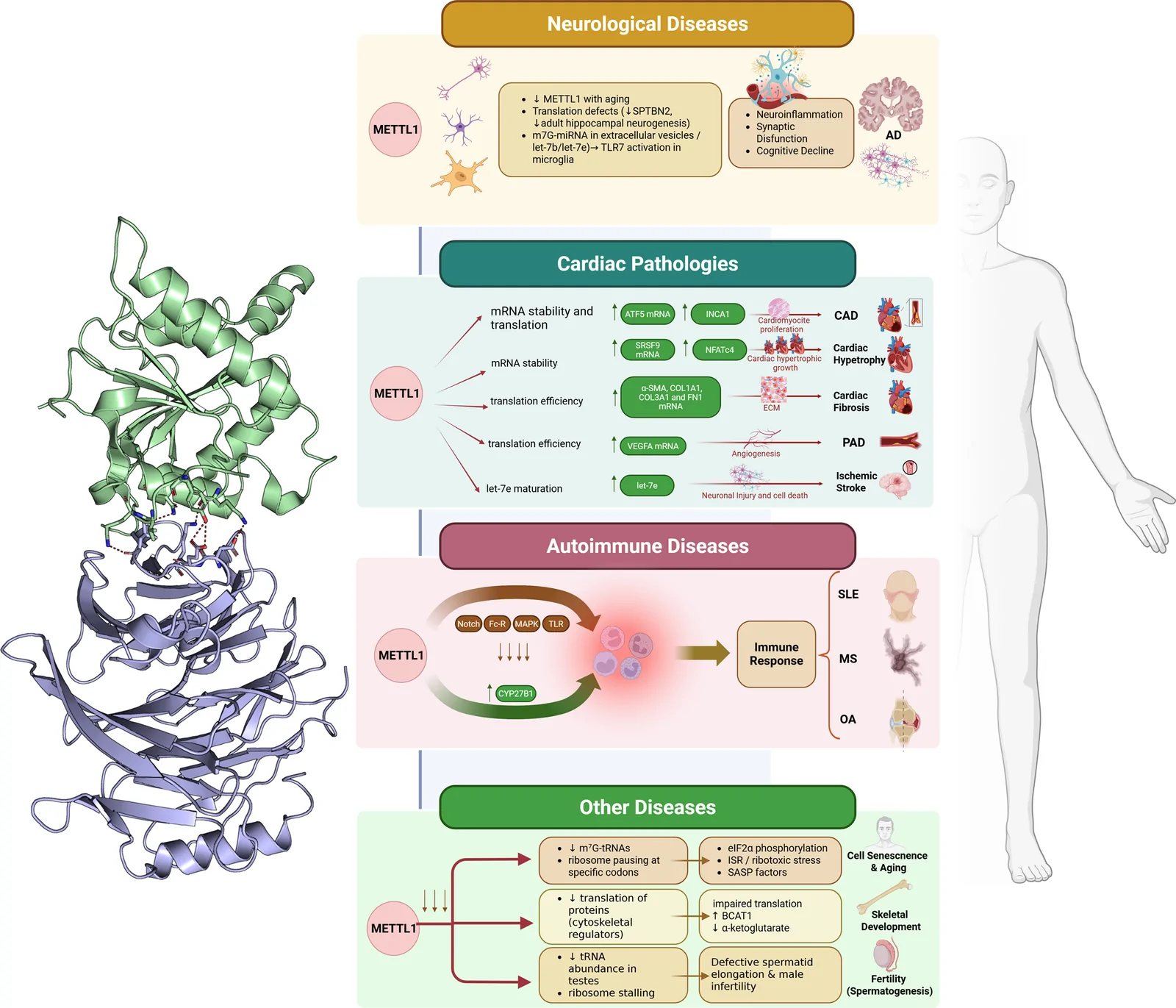
Role of m^7^G modification in neurological, cardiovascular, autoimmune, and other diseases. (Created in https://BioRender.com).

### METTL1 in Neurodevelopment and Neurodegenerative Diseases

Neurodevelopment and neurodegeneration are exquisitely sensitive to perturbations in translational control, and accumulating evidence identifies METTL1/WDR4-mediated m^7^G as a critical regulator of neural homeostasis across the lifespan (Figure 2). During early brain development, METTL1/WDR4-dependent m^7^G modification is indispensable for proper neural specification and growth. Loss-of-function mutations in WDR4 cause microcephalic primordial dwarfism, a severe neurodevelopmental disorder characterized by profound microcephaly, growth retardation, epileptic encephalopathy, and global depletion of tRNA m^7^G. Complementary experimental models demonstrate that METTL1 silencing disrupts neurectoderm induction and early neural lineage commitment, underscoring the requirement for intact m^7^G machinery during the earliest stages of neurogenesis.^25^ Mechanistically, m^7^G loss selectively impairs translation of long, codon-biased developmental transcripts rather than globally suppressing protein synthesis. Core neurodevelopmental regulators such as OTX2, SOX1, and PAX6 are particularly dependent on m^7^G-modified tRNAs for efficient decoding. When METTL1/WDR4 activity is compromised, protein output from these transcripts is markedly reduced despite the mRNA abundance is preserved. In parallel, METTL1 regulates neural fate decisions through m^7^G-dependent processing of pri–let-7 microRNAs. Let-7 family members orchestrate neurogenesis by repressing HMGA2, LIN28, and ASCL1. Defective let-7 maturation contributes to aberrant neurogliogenesis and impaired function of the adult neurogenic niche.^25^

Microcephalic primordial dwarfism refers to a collection of rare inherited neurodevelopmental conditions caused by single-gene defects that lead to marked reductions in both brain and body growth starting early in development. Common features include pronounced prenatal and postnatal growth failure, microcephaly, distinctive facial characteristics, and an unusually sociable or pleasant demeanor. Whole-exome sequencing performed on two Egyptian patients with MPD revealed a previously unreported missense variant in the WDR4 gene, which was implicated as the underlying cause. In humans, pathogenic variants in WDR4 give rise to a unique MPD phenotype characterized by facial abnormalities, structural brain defects, and severe encephalopathy with seizures.^21,26^ In Down syndrome, hippocampal WDR4 expression is reduced in both mouse models and human transcriptomic data sets. Restoration of WDR4 expression enhances synaptic plasticity and rescues learning and memory deficits, implicating m^7^G-dependent translational control as a modulator of cognitive dysfunction in trisomy 21.^27–29^

Beyond development, altered METTL1 and WDR4 expression has been reported across multiple neurodegenerative disorders, suggesting a broader role for m^7^G dysregulation in neuronal vulnerability. Alzheimer’s disease (AD), the most common irreversible form of dementia, is characterized by progressive cognitive dysfunction and pronounced neuronal loss, which has been linked to impaired adult hippocampal neurogenesis.^30^ In AD, single-cell RNA sequencing from mouse models reveals reduced METTL1 expression across excitatory neurons, astrocytes, and microglia. Notably, METTL1 expression and global m^7^G modification levels are significantly higher in differentiated neurons than in neural stem cells, and METTL1 overexpression promotes neuronal differentiation and proliferation. A mechanistic link between m^7^G and neurodegeneration is suggested by studies of CD47-mediated extracellular vesicle loading of m^7^G-modified microRNAs,^31^ including pro-inflammatory let-7 species. These let-7 miRNAs activate toll-like receptor 7 (TLR7) signaling in microglia, driving neuroinflammation and neuronal injury; notably, elevated cerebrospinal fluid levels of let-7b and let-7e have been detected in patients with AD.^32–36^ Mechanistically, METTL1-mediated internal m^7^G modification enhances expression of the cytoskeletal protein SPTBN2 by increasing SPTBN2 mRNA stability and translational efficiency, thereby promoting hippocampal neurogenesis. Consistent with this role, METTL1 knockdown impairs adult hippocampal neurogenesis and spatial memory, whereas METTL1 overexpression rescues defective neurogenesis and cognitive deficits in adult mouse models of AD.^37,38^ Finally, in Parkinson’s disease (PD), Huntington’s disease (HD), and amyotrophic lateral sclerosis (ALS), disease-specific transcriptomic studies consistently report reduced METTL1 expression or alterations in m^7^G-modified tRNA and miRNA species. These changes are predicted to affect neuronal stress responses, synaptic maintenance, ribosome dynamics, and survival signaling, positioning m^7^G dysregulation as a convergent epitranscriptomic node across neurodegenerative pathways.^25^

### METTL1 in Cardiovascular Diseases

Cardiovascular diseases (CVDs), encompassing coronary artery disease (CAD), heart failure (HF), cardiomyopathies, pulmonary arterial hypertension (PAH), ischemic stroke, and peripheral arterial disease (PAD),^39^ remain the leading cause of global morbidity and mortality. Accumulating evidence suggests that METTL1/WDR4-mediated m^7^G remodeling plays context-dependent roles across diverse cardiovascular pathologies (Figure 2).^40,41^

In CAD, genome-wide association studies have linked multiple m^7^G-related single-nucleotide polymorphisms to disease susceptibility. Notably, variants mapping to METTL1-regulated gene networks, including an m^7^G-associated polymorphism within the long noncoding RNA PVT1, have reached genome-wide significance. Although direct mechanistic links between METTL1 activity and atherosclerotic plaque development remain to be fully elucidated, these genetic data implicate perturbations of m^7^G homeostasis as contributors to coronary risk.^42–44^

A more direct pathogenic role for METTL1 has been demonstrated in cardiac hypertrophy. In mouse models of pressure overload and neurohumoral stimulation, METTL1 expression and global m^7^G abundance are markedly increased in hypertrophied myocardium. This upregulation is predominantly observed in cardiomyocytes and correlates with disease severity, suggesting a cell-autonomous contribution of METTL1 to hypertrophic signaling.^45^ Mechanistically, METTL1 promotes m^7^G methylation of SRSF9 mRNA, enhancing its translation and driving an alternative splicing program that stabilizes NFATc4, a central transcriptional regulator of maladaptive hypertrophic growth. SRSF9-dependent splicing selectively favors pro-hypertrophic transcript isoforms, reinforcing sustained NFAT signaling and transcription of fetal gene programs associated with cardiac dysfunction.^45^ Consistent with this mechanism, METTL1 overexpression exacerbates ventricular remodeling, whereas genetic ablation of METTL1 attenuates pathological hypertrophy, highlighting the METTL1–SRSF9–NFATc4 axis as a candidate therapeutic target.^45^

In cardiac fibrosis, METTL1 emerges as a key regulator of extracellular matrix production following myocardial infarction. Cardiac fibrosis represents a common pathological feature of diverse heart diseases and contributes to both mechanical stiffness and electrical dysfunction of the myocardium, ultimately predisposing to heart failure and arrhythmias.^46^ Fibroblast-specific deletion of *Mettl1* significantly reduces collagen deposition and improves ventricular architecture. At the molecular level, METTL1 was suggested to methylate mRNAs encoding α-smooth muscle actin, collagen Iα1, collagen IIIα1, and fibronectin 1, increasing their translational efficiency and reinforcing the myofibroblast phenotype that underlies fibrotic remodeling.^47,48^ Consistently, METTL1-mediated m^7^G modification is markedly elevated in fibrotic cardiac tissue, and TGF-β1–induced activation and *trans*-differentiation of cardiac fibroblasts into myofibroblasts are accompanied by enhanced METTL1-dependent m^7^G methylation. Conversely, fibroblast-specific ablation of METTL1 reduces the abundance and translation of m^7^G-modified profibrotic transcripts, resulting in attenuation of myocardial infarction–induced heart failure and fibrosis.^47^

In contrast, METTL1 appears to play a protective role in PAD. In hind-limb ischemia models, ischemic injury is associated with reduced METTL1 expression and diminished global m^7^G levels. Restoration of METTL1 expression enhances perfusion recovery, capillary density, and arteriogenesis, at least in part through direct m^7^G methylation of VEGFA mRNA and subsequent amplification of pro-angiogenic signaling. These findings underscore the context-dependent nature of METTL1 function and suggest that selective activation of METTL1 may be beneficial in ischemic vascular disease.^49,50^

In HF, elevated METTL1 expression has been detected in human failing myocardium, whereas cardiomyocyte-specific METTL1 deficiency confers protection against postinfarction systolic dysfunction in mouse models. METTL1-driven m^7^G remodeling influences inflammatory cell infiltration and cytokine signaling within the failing heart, although the precise molecular circuits involved remain incompletely defined.^51,52^ Beyond its contribution to established HF, METTL1 critically regulates cardiomyocyte fate following myocardial infarction, a central pathological event driving adverse cardiac remodeling. METTL1 has been reported to enhance m^7^G modification of ATF5 mRNA, leading to elevated ATF5 expression and subsequent transcriptional activation of INCA1, a CDK2 inhibitor that interacts with cyclin A1. This regulatory cascade induces cell-cycle arrest at the S to G2/M transition, thereby limiting cardiomyocyte proliferation and impairing cardiac regenerative capacity.^53^ In addition, METTL1 promotes the stability of cylindromatosis (CYLD) mRNA through m^7^G methylation, resulting in increased CYLD protein levels. Elevated CYLD activity enhances P53 deubiquitination, ultimately facilitating cardiomyocyte apoptosis during ischemia/reperfusion injury.^54^

Emerging genetic and transcriptomic data also implicate m^7^G pathways in PAH, where m^7^G-associated polymorphisms correlate with blood pressure traits and m^7^G-dependent long noncoding RNA signatures associate with immune microenvironment alterations. However, direct causal roles of METTL1/WDR4 in pulmonary vascular remodeling require further investigation.^55,56^

Ischemic stroke is the most common cerebrovascular disorder and remains a leading cause of mortality and long-term disability worldwide, a burden that continues to increase with population aging.^57^ Recently, epitranscriptomic regulation has emerged as an important contributor to stroke pathophysiology. Dysregulated m^6^A modification has been linked to ischemic injury,^58–60^ and accumulating evidence now implicates m^7^G as an additional regulatory layer. Transcriptomic analyses of patient data sets revealed significant dysregulation of multiple m^7^G-regulated genes in ischemic stroke, several of which were validated in experimental middle cerebral artery occlusion models, supporting a functional association between altered m^7^G pathways and ischemic brain injury.^61^ Because ischemia and hypoxia are central drivers of neuronal death,^62^ the observation that m^7^G modifications are present on brain mRNAs under both normoxic and hypoxic conditions is particularly relevant. Hypoxia-induced changes in m^7^G methylation preferentially affect transcripts involved in Notch signaling,^63^ a pathway known to promote neuronal apoptosis through interactions with p53, NF-κB, HIF-1α, and c-Jun during cerebral ischemia.^63–66^ Noncoding RNA regulation provides an additional mechanistic link, as elevated levels of the pro-neurotoxic miRNA let-7e have been detected in the serum and cerebrospinal fluid of ischemic stroke patients,^67^ and let-7 family members exacerbate neuronal injury in experimental models.^68^ Given that METTL1 catalyzes the m^7^G modification of pri-let-7e, altered METTL1 activity may influence stroke outcomes by modulating let-7 biogenesis. Consistently, disruption of the METTL1 cofactor WDR4 destabilizes METTL1, impairs tRNA m^7^G modification, and induces neuronal apoptosis, whereas restoration of WDR4 rescues neuronal survival.^26,69^ Notably, METTL1 has also been shown to exacerbate myocardial ischemia-reperfusion injury through m^7^G methylation of CYLD mRNA, highlighting a conserved role of METTL1-mediated m^7^G remodeling in ischemic injury across cardiovascular and cerebrovascular tissues. Moreover, knockdown of METTL1 and pharmacological inhibition of methyltransferase activity achieved with sinefungin (a broadly acting, nonselective SAM analog) were also associated with reduced infarct size. While this supports a therapeutic rationale, the pharmacological evidence should be interpreted cautiously, as sinefungin does not specifically inhibit METTL1 and may confound efficacy through off-target methyltransferase blockade.^54^

### METTL1 in Autoimmune Diseases

Emerging evidence indicates that the METTL1/WDR4 complex plays a significant role in selected autoimmune diseases (Figure 2). Systemic lupus erythematosus (SLE) represents a paradigmatic systemic autoimmune disorder marked by the generation of pathogenic autoantibodies and widespread organ involvement, arising from profound alterations in immune cell phenotype and function. Although immunosuppressive regimens have improved disease management, their limited specificity and frequent association with serious adverse events, such as infections and irreversible organ injury, highlight the unmet need for more selective and personalized therapeutic approaches.^70^

Recent transcriptomic profiling studies have identified a significant downregulation of METTL1 expression in patients with SLE. Reduced METTL1 levels are positively associated with diminished peripheral B-cell populations while inversely correlating with suppressive T-cell subsets, suggesting that impaired METTL1 activity may contribute to immune dysregulation during disease progression. Moreover, METTL1 expression shows an inverse relationship with several immune and inflammatory signaling pathways central to SLE pathogenesis, including Notch signaling, Fc receptor-dependent phagocytosis, mitogen-activated protein kinase (MAPK) cascades, and Toll-like receptor pathways, all of which critically regulate immune activation and cytokine production.^71,72^

Consistent with this notion, METTL1 expression positively correlates with activated CD4^+^ and CD8^+^ T-cell subsets, which are known to exhibit aberrant activation and signaling in SLE. Dysregulated T-cell responses amplify inflammatory signaling and promote pathological B-cell activation and autoantibody production.^38,71,72^

Multiple sclerosis (MS) is a chronic autoimmune disease characterized by sustained neuroinflammation, demyelination, and progressive neuronal loss.^73^ Genetic association studies have implicated METTL1 as a susceptibility factor in MS. In particular, tag-single nucleotide polymorphism analyses identified a functional variant (rs10877013) within the 12q13.3–12q14.1 locus that alters enhancer activity in an allele- and orientation-dependent manner.^74^ This variant modulates long-range chromatin interactions, thereby influencing the transcriptional regulation of genes within the KIF5A–CYP27B1–METTL1–FAM119B genomic region.^38,75^

The involvement of METTL1 in MS pathogenesis is further supported by its connection to vitamin D metabolism, a well-established environmental risk factor for the disease. CYP27B1, a parathyroid hormone–inducible enzyme essential for vitamin D activation, has been proposed as a key causal gene within this locus. Experimental evidence indicates that METTL1 deficiency disrupts parathyroid hormone–mediated induction of CYP27B1, leading to impaired vitamin D bioactivation and altered immune regulation.^38,75,76^

Osteoarthritis (OA), the most prevalent form of arthritis worldwide and a leading cause of disability, lacks effective disease-modifying therapies. Emerging bioinformatic and transcriptomic analyses have revealed a marked reduction of METTL1 expression in OA cartilage relative to healthy tissue. Notably, METTL1 levels strongly correlate with patterns of immune cell infiltration and immune-related functional pathways within the osteoarthritic microenvironment.^77^ Furthermore, METTL1 has been identified as a central m^7^G-associated hub gene during OA progression and has been incorporated into predictive models for disease occurrence. These findings implicate METTL1-mediated m^7^G regulation in OA pathophysiology, potentially through modulation of inflammatory responses and immune infiltration, and provide a molecular rationale for exploring METTL1 as a target for innovative immunomodulatory strategies in osteoarthritis.^38,78^

### METTL1 in Aging, Skeletal Disorders, and Reproductive Function

METTL1-catalyzed m^7^G modification of tRNAs is fundamental for preserving the fidelity of protein synthesis and proteome integrity, with consequential impacts on cellular aging and senescence (Figure 2). Loss of METTL1 expression and concomitant decline in m^7^G-modified tRNAs are features of replicative and organismal aging, reflecting a progressive reduction in METTL1/WDR4 activity at both the transcriptional and protein levels. This deficit undermines the stability of a subset of tRNAs and facilitates their rapid degradation, ultimately decreasing tRNA pools essential for elongation. The resulting ribosome pausing at codons decoded by m^7^G-deficient tRNAs impairs global translational efficiency, preferentially diminishing the synthesis of proteins involved in key processes such as WNT signaling and ribosome biogenesis. Persistent translational stress engages ribotoxic and integrated stress responses, including eIF2α phosphorylation and activation of stress kinase pathways, which promote a senescence-associated secretory phenotype and other hallmarks of cellular aging. Restoration of translation elongation factors such as eEF1A partially mitigates these senescence phenotypes, underscoring the mechanistic link between m^7^G-dependent translation and aging phenotypes.^38,53,79^

Beyond its role in aging, METTL1 also contributes to skeletal morphogenesis (Figure 2). The m^7^G modification of tRNAs by METTL1 is required for efficient translation of mRNAs encoding cytoskeletal regulators and components of Rho GTPase signaling, which are essential for mesenchymal progenitor proliferation and osteogenic commitment. Conditional deletion of *Mettl1* or disease-associated mutations in its cofactor *Wdr4* severely compromise endochondral bone formation, resulting in impaired longitudinal growth and reduced bone mass accrual in mice. In these models, loss of m^7^G-modified tRNAs is accompanied by upregulation of branched-chain amino acid transaminase 1 (BCAT1) and a metabolic rewiring that restricts intracellular α-ketoglutarate, linking translational defects to disrupted cellular metabolism. Notably, supplementation with α-ketoglutarate partially rescues skeletal defects, suggesting that metabolic interventions may ameliorate the consequences of m^7^G dysregulation in primordial dwarfism and related skeletal dysplasias.^80^

In reproductive biology, the requirement for METTL1-mediated tRNA modification is exemplified by its essential role in spermatogenesis (Figure 2). Studies in *Drosophila* demonstrate that deletion of the METTL1 ortholog abolishes m^7^G modification on a subset of tRNAs and decreases their abundance specifically in testes, leading to ribosome stalling at corresponding codons and inefficient translation of transcripts critical for spermatid elongation and sperm stability. This translational defect results in male infertility, while germ cell-specific restoration of METTL1 rescues both tRNA modification and fertility, highlighting an evolutionarily conserved function of METTL1 in germ cell development.^38,81^

METTL1/WDR4 in Cancer: Mechanisms, Biological Roles, and Tumor Contexts.

### Mechanisms of METTL1/WDR4 in Cancer

m^7^G has recently emerged as a central epitranscriptomic modification with a profound impact on malignant transformation and tumor progression.^82^ Across multiple cancer types, aberrant METTL1 and/or WDR4 expression correlates with aggressive disease, metastasis, and poor clinical outcome, reflecting their central role in shaping oncogenic gene expression programs.^83^

The primary oncogenic mechanism of METTL1/WDR4 is tRNA-centered. The complex catalyzes m^7^G modification at position 46 in the variable loop of a defined subset of tRNAs. This modification enhances tRNA structural stability, correct folding, and decoding efficiency, thereby expanding the pool of translationally competent tRNAs. Elevated m^7^G-modified tRNA levels reduce ribosome pausing at cognate codons and selectively enhance translation of mRNAs enriched for those codons. Importantly, METTL1/WDR4 does not globally increase protein synthesis, but instead reprograms translation in a codon-bias–dependent manner, preferentially amplifying oncogenic transcripts encoding cell-cycle regulators, growth factor signaling components, and metabolic enzymes. Increased levels of individual m^7^G-modified tRNAs, such as Arg-TCT tRNAs, is sufficient to promote malignant transformation, underscoring the causal role of tRNA m^7^G in oncogenesis.^22,82–85^

A second layer of oncogenic activity involves METTL1-dependent internal m^7^G modification of mRNAs. Although less widespread than tRNA methylation, internal m^7^G marks on selected mRNAs increase transcript stability and translational efficiency, reinforcing oncogenic pathways. These mRNA-centric effects synergize with the tRNA codon-bias mechanism: m^7^G-modified tRNAs enhance decoding, while m^7^G-modified mRNAs are preferentially stabilized and translated, resulting in selective amplification of defined pro-tumor gene networks rather than indiscriminate translational upregulation.^83,86^

Third, METTL1/WDR4 influences noncoding RNA processing and stress-adaptive translation. The deposition of m^7^G can facilitate the maturation of structured pri-miRNAs by alleviating inhibitory secondary structures, in turn reshaping miRNA networks that control proliferation, migration, and invasion.

In parallel, METTL1/WDR4 supports translational adaptation to oncogenic stress by maintaining tRNA integrity and translational capacity under nutrient limitation or hypoxia. Through these mechanisms, METTL1/WDR4 enhances tumor plasticity, therapy resistance, and survival in hostile microenvironments.^83,87,88^

These three interconnected layers, tRNA, mRNA, and noncoding RNA/stress pathways, converge on fundamental malignant phenotypes including uncontrolled proliferation, increased invasiveness, metabolic plasticity, therapy resistance, and immune evasion. Across many tumor types, aberrant expression of METTL1 and/or WDR4, and corresponding alterations in m^7^G levels, correlate with advanced stage, metastasis, and adverse clinical outcomes. m^7^G-related gene signatures associate with immune infiltration patterns and responsiveness to chemotherapy or targeted agents, suggesting that m^7^G remodeling stratifies tumors into functionally distinct epitranscriptomic states.^83,86^

Although the RNMT–RAM cap methyltransferase complex also contributes to oncogenic signaling by increasing the m^7^G cap on specific transcripts such as Cyclin D1, particularly under the influence of c-MYC, its role remains less clearly defined than that of METTL1/WDR4. The METTL1 axis stands out for its ability to reprogram codon-biased translation and stabilize distinct mRNA subsets, providing a mechanistic framework directly amenable to small-molecule inhibition strategies.^86^

### Biological Roles of METTL1 in Cancer

Accumulating evidence highlights METTL1 as a pivotal regulator of tumor initiation and progression via m^7^G-dependent translational programs. Aberrant METTL1’s activity contributes to multiple hallmarks of cancer, including uncontrolled proliferation, metastasis, angiogenesis, chemoradiotherapy resistance, metabolic reprogramming, and suppression of the tumor immune microenvironment (TIME) (Figure 3).^38^

**Figure 3:**
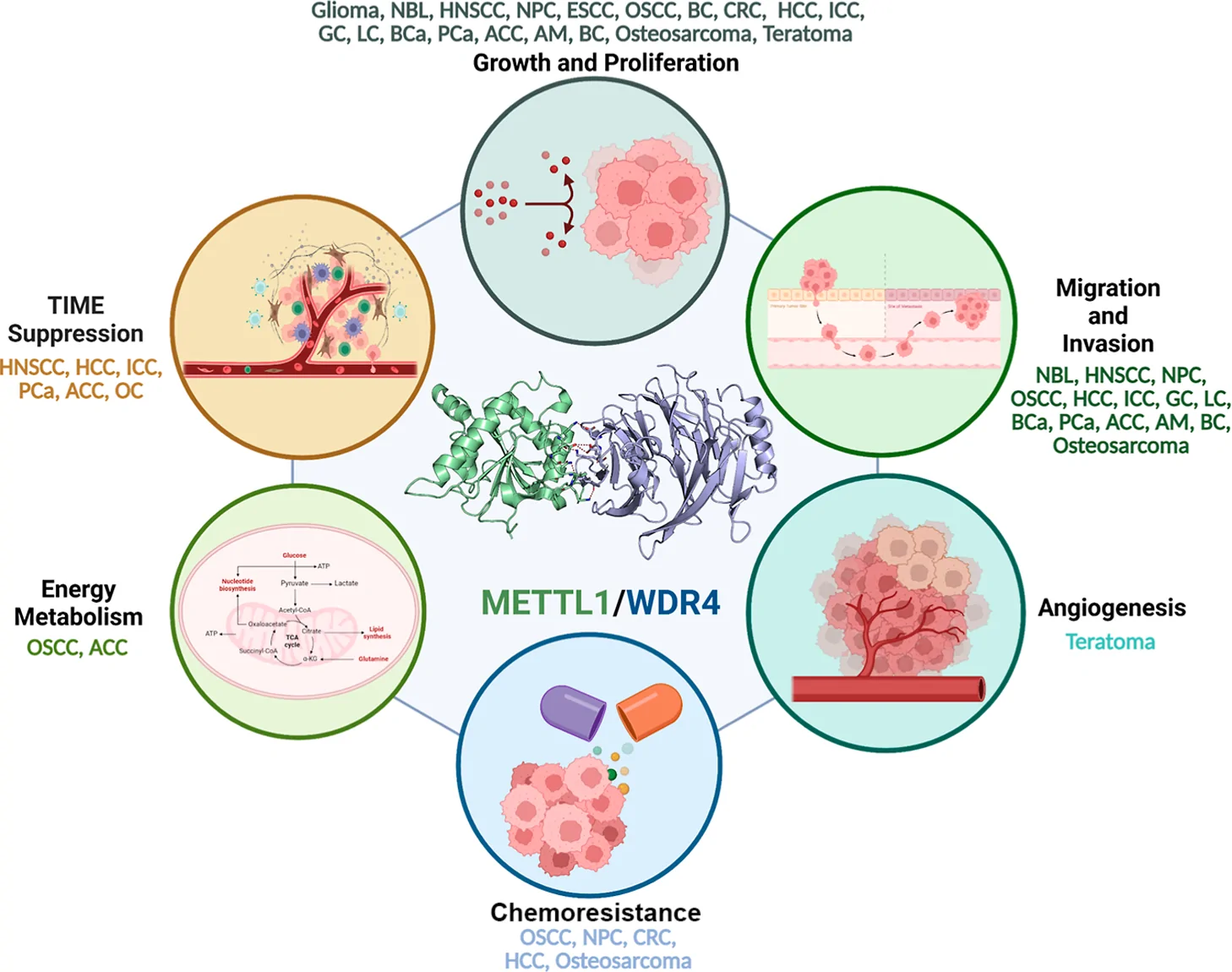
Overview of the biological roles of the METTL1/WDR4 complex in cancer. METTL1/WDR4 dysregulation has been implicated in multiple hallmarks of tumor progression, including cancer cell growth and proliferation, migration and invasion, angiogenesis, chemoresistance, altered energy metabolism, and immune suppression. For each functional axis, the figure highlights the specific tumor types in which aberrant METTL1/WDR4 activity has been reported. (Created in https://BioRender.com).

A central role of METTL1 in cancer is the regulation of cell-cycle progression and proliferation. METTL1 promotes tumor growth by modulating key cell-cycle regulators in diverse malignancies. In colorectal cancer (CRC), METTL1 attenuates CHEK2-mediated G1/S checkpoint arrest, enhancing proliferation.^89^ In HCC, METTL1 depletion reduces cyclin A2 translation, inducing G2/M arrest. It also indirectly supports proliferation of castration-resistant prostate cancer cells via CDK14 signaling.^90^ Context-dependent effects exist: the METTL1–ATF5–INCA1 axis inhibits cardiomyocyte proliferation through CDK2–cyclin A1 disruption, causing S–G2/M arrest,^44^ while in breast cancer, METTL1 suppresses proliferation by enhancing translation of GADD45A and RB1, selectively blocking G2/M progression.^91^

METTL1 further promotes tumor aggressiveness by enhancing migration and invasion, processes responsible for most cancer-related mortality. Loss of METTL1 impairs migratory and invasive capacities in HCC, whereas overexpression enhances them. Similar pro-metastatic effects are documented in osteosarcoma, lung cancer, bladder cancer, nasopharyngeal carcinoma, and oral squamous cell carcinoma.^38^

Angiogenesis is another METTL1-regulated process, critical for tumor growth and metastasis. METTL1 silencing biases human-induced pluripotent stem cells toward mesodermal differentiation while suppressing neuroectodermal fate, enhancing angiogenic potential and teratoma formation. METTL1-deficient hiPSC-derived endothelial progenitors stimulate vascular smooth muscle proliferation in coculture. In ischemic contexts, METTL1 promotes angiogenesis by enhancing VEGFA mRNA translation in an m^7^G-dependent manner.^92^

METTL1 also modulates resistance to chemotherapy and radiotherapy. In oral squamous cell carcinoma, METTL1 increases anlotinib resistance by enhancing translation of respiratory chain enzymes and oxidative phosphorylation.^93^ It promotes doxorubicin resistance in osteosarcoma via oncogenic mRNA translation.^14^ Conversely, the METTL1/pri-miR-26a/FTH1 axis induces ferroptosis and chemosensitivity in osteosarcoma, highlighting context-dependent effects. In HCC, METTL1 enhances lenvatinib resistance through EGFR pathway translation and facilitates DNA double-strand break repair, promoting radioresistance.^94^

METTL1 drives metabolic reprogramming, promoting glycolysis in adrenocortical carcinoma via HK1 upregulation.^95^ In anlotinib-resistant OSCC, METTL1 supports oxidative phosphorylation, whereas knockdown reduces oxidative phosphorylation (OXPHOS) complex expression and ROS production.^93^

Finally, METTL1 shapes the so-called TIME. It enhances m^7^G-dependent translation of CXCL5 and CXCL8, recruiting myeloid-derived suppressor cells and promoting immunosuppression in HCC and intrahepatic cholangiocarcinoma. High METTL1 correlates with reduced CD8^+^ T-cell infiltration and increased M2 macrophages. METTL1 regulates tumor-associated macrophage polarization, favoring an M2 immunosuppressive phenotype, whereas its inhibition shifts macrophages toward an M1-like state in prostate cancer models.^38,96^

### METTL1/WDR4: Tumor-specific Insights

The functional impact of METTL1/WDR4 in cancer is Janus-faced, as it possesses context-dependent roles, acting either as a tumor-promoting or tumor-suppressive factor depending on the cellular and molecular landscape. In the following sections, we discuss individually the major cancer types in which METTL1/WDR4 dysregulation has been reported, highlighting both convergent and divergent mechanisms of action. These findings are systematically summarized in Figure 4 and Table 1.

**Figure 4:**
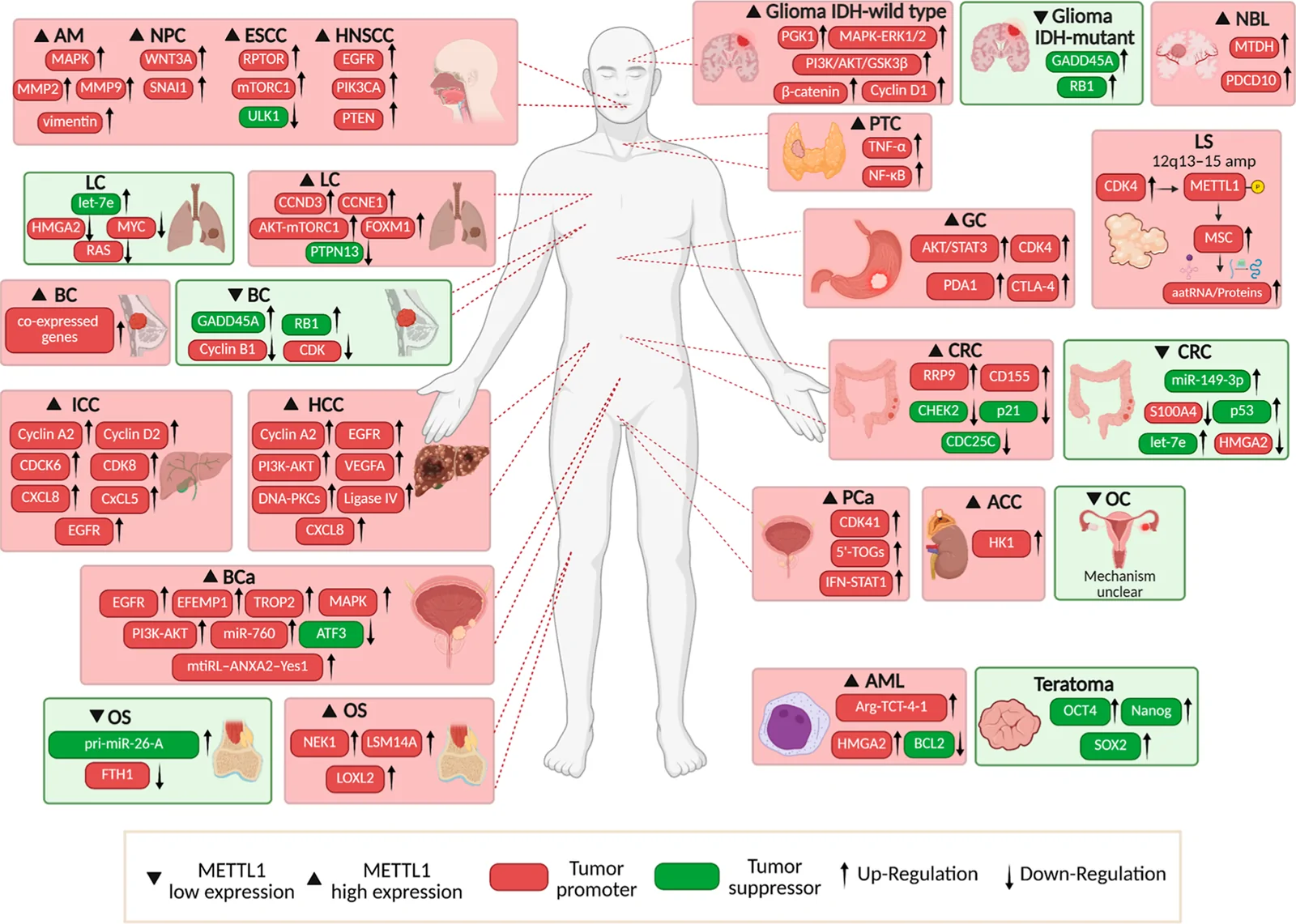
Regulation of METTL1/WDR4 across human cancers. Light red panels indicate tumor types in which METTL1 predominantly functions as a tumor promoter, whereas light green panels denote cancers in which METTL1 exhibits a tumor-suppressive role. Within each tumor-specific panel, red boxes denote oncogenic RNAs, proteins, regulatory factors, or signaling pathways, while green boxes indicate oncosuppressive RNAs, proteins, factors, or pathways. Upward and downward arrows indicate relative up- or downregulation, respectively. Cancer types and abbreviations are listed in alphabetical order: ACC, adrenocortical carcinoma; AM, ameloblastoma; AML, acute myeloid leukemia; BC, breast cancer; BCa, bladder cancer; CRC, colorectal cancer; ESCC, esophageal squamous cell carcinoma; GC, gastric cancer; HCC, hepatocellular carcinoma; HNSCC, head and neck squamous cell carcinoma; ICC, intrahepatic cholangiocarcinoma; LC, lung cancer; LS, liposarcoma; NBL, neuroblastoma; NPC, nasopharyngeal carcinoma; OC, ovarian cancer; OS, osteosarcoma; PC, prostate cancer; PTC, papillary thyroid carcinoma. (Created in https://BioRender.com).

**Table 1:** Different Oncogenic/Oncosuppressive Roles of METTL1/WDR4 and Related Mechanisms in Cancer

cancer type	cell lines	role	m^7^G target	functional effects	mechanism/pathway	ref
**glioma**	U87 (IDH-wild type)	**tumor promoter**	tRNA	proliferation ↑	•METTL1 tRNA m^7^G modification enhances decoding of growth-promoting transcripts	^97–100^
					•activates MAPK/ERK1/2 signaling	
					•stabilizes metabolic transcripts (PGK1), supporting glycolysis	
					•PI3K/AKT/GSK3β and β-catenin pathways are also activated and Cyclin D1 is upregulated	
	IDH mutant	**tumor suppressor**	tRNA	G2/M arrest ↑, proliferation ↓	•METTL1 tRNA m^7^G enhances translation of checkpoint regulators GADD45A and RB1	^101,102^
					•strengthens G2/M checkpoint, constraining tumor progression	
					•loss of METTL1 shortens G2/M and reduces genomic stability	
**NBL**	KELLY, BE2C	**tumor promoter**	tRNA	proliferation ↑, migration ↑, apoptosis ↓	•METTL1 tRNA m^7^G enhances translation of oncogenic targets MTDH and PDCD10	^103^
					•promotes global translational efficiency, sustaining protein synthesis	
**HNSCC**	SCC9, SCC15	**tumor promoter**	tRNA	proliferation ↑, migration ↑, invasion ↑, apoptosis ↓, TIME ↓	•METTL1/WDR4 overexpression increases tRNA m^7^G modification	^105^
					•enhances codon-biased translation of oncogenes (e.g., EGFR, PIK3CA, PTEN)	
					•activates PI3K–AKT–mTOR signaling, promoting proliferation, EMT, and survival	
					•contributes to immunosuppressive tumor microenvironment	
**ESCC**	KYSE150, KYSE30	**tumor promoter**	tRNA	growth ↑, proliferation ↑, autophagy ↓	•METTL1 catalyzes tRNA m^7^G, enhancing translation of RPTOR	^104^
					•sustains mTORC1 signaling and inhibits ULK1-driven autophagy	
					•loss of METTL1 reduces RPTOR, attenuates mTORC1, and increases autophagy, reducing tumor aggressiveness	
**NPC**	5–8F, 6–10B	**tumor promoter**	tRNA	growth ↑, proliferation ↑, migration ↑, invasion ↑, apoptosis ↓, EMT ↑, chemotherapy resistance ↑	•ARNT-driven METTL1/WDR4 overexpression enhances tRNA m^7^G modification	^106–108^
					•increases translation of WNT3A, stabilizing SNAI1, Cyclin D1, c-Myc	
**OSCC**	SCC9, SCC15	**tumor promoter**	tRNA	growth ↑, proliferation ↑, migration ↑, glycolysis/OXPHOS ↑, chemotherapy resistance ↑	•METTL1 overexpression increases tRNA m^7^G pools, enhancing translational output	^93^
					•boosts metabolic enzymes, shifting to OXPHOS (oxidative phosphorylation)	
					•upregulates growth regulators, promoting proliferation and migration	
					•supports chemotherapy resistance (anlotinib)	
**AM**	hTERT-AM	**tumor promoter**	tRNA	growth ↑, proliferation ↑, migration ↑, invasion ↑	•METTL1 tRNA m^7^G increases translation of MAPK pathway effectors	^109^
					•upregulates Cyclin D1, MMP2, MMP9, and vimentin	
					•promotes cell-cycle progression, extracellular matrix remodeling, and EMT	
**PTC**	-	**tumor promoter**	tRNA	proliferation ↑, migration ↑, metastasis ↑	•METTL1 tRNA m^7^G modification increases translation of codon-biased mRNAs (e.g., TNF-α)	^110^
					•enhances NF-κB signaling, promoting proliferation and metastasis	
					•cooperates with WDR4 to stabilize oncogenic transcripts	
					•knockdown reduces translation of TNF-α, impairing NF-κB signaling	
**LC**	A549, NCI-H1299	**tumor promoter**	tRNA	growth ↑, proliferation ↑, migration ↑, invasion ↑	•METTL1/WDR4 tRNA m^7^G increases translation of CCND3 and CCNE1	^111^
					•activates AKT–mTORC1, promoting proliferation, invasion, and xenograft growth	
	A549	**tumor suppressor**	miRNA	migration ↓	•METTL1 m^7^G facilitates maturation of let-7e miRNA	^11^
					•let-7e represses HMGA2, inhibiting EMT and migration	
**GC**	AGS, HGC-27	**tumor promoter**	tRNA/unspecified	proliferation ↑, migration ↑, invasion ↑, apoptosis ↓	•METTL1 overexpression increases tRNA m^7^G modification and possibly coregulates nearby oncogene CDK4	^112,113^
					•enhances translation of proliferation- and migration-related genes	
					•activates AKT/STAT3 pathways	
					•contributes to immune evasion by upregulating PD-1 and CTLA-4	
**HCC**	Huh7, MHCC97H, HepG2	**tumor promoter**	tRNA	proliferation ↑, migration ↑, invasion ↑, apoptosis ↓	•METTL1/WDR4 tRNA m^7^G increases translation of Cyclin A2, EGFR, and VEGFA	^23,114^
					•activates PI3K–AKT and MAPK pathways	
					•enhances EMT and metastatic potential	
					•suppresses PTEN signaling	
	MHCC97H, SNU449	**tumor promoter**	tRNA	radiotherapy resistance ↑, TIME ↓	•METTL1 tRNA m^7^G increases translation of DNA-PKcs and DNA ligase IV	^115,117^
					•enhances NHEJ-mediated DNA double-strand break repair	
					•stimulates TGF-β2–PMN-MDSC axis	
	Huh7, PLC/PRF/5	**tumor promoter**	tRNA	chemotherapy resistance ↑	•METTL1 tRNA m^7^G increases EGFR translation under lenvatinib treatment	^94,116^
					•Supports adaptive resistance to targeted therapy	
**ICC**	HuCCT1, RBE	**tumor promoter**	tRNA	growth ↑, migration ↑, invasion ↑, apoptosis ↓	•METTL1/WDR4 tRNA m^7^G establishes a codon-biased translational landscape	^119^
					•enhances translation of Cyclin A2, D2, CDK6/8, and EGFR pathway components	
					•Suppresses apoptosis	
	HuCCT1, RBE	**Tumor Promoter**	tRNA	TIME ↓, immunotherapy resistance ↑	•METTL1 enhances translation of CXCL8/Cxcl5	^121^
					•Promotes recruitment of CXCR2^+^ PMN-MDSCs	
					•generates an immunosuppressive microenvironment, reducing the efficacy of PD-1 blockade	
**CRC**	HCT116, RKO	**tumor promoter**	tRNA	proliferation ↑, growth ↑	•METTL1 tRNA m^7^G enhances translation of pro-proliferative transcripts	^89,121^
					•suppresses checkpoint kinase CHEK2 and reduces p21 and CDC25C phosphorylation	
					•Accelerates G1/S and G2/M transitions	
	HCT116	**tumor suppressor**	miRNA	chemotherapy sensitivity ↑	•METTL1 m^7^G promotes miR-149–3p maturation, represses S100A4, and stabilizes p53, enhancing apoptosis and cisplatin sensitivity	^122,123^
					•METTL1 m^7^G facilitates maturation of let-7e miRNA and let-7e represses HMGA2, inhibiting EMT and migration	
**BCa**	T24, UMUC-3	**tumor promoter**	tRNA	proliferation ↑, migration ↑, invasion ↑	•METTL1 tRNA m^7^G enhances translation of EGFR and EFEMP1	^124–126^
					•Activates PI3K–AKT and MAPK signaling	
	T24, UMUC-3	**tumor promoter**	miRNA	proliferation ↑, migration ↑	•METTL1 mediates m^7^G processing of pri-miR-760	^124–127^
					•represses ATF3, promoting expression of TROP2	
					•enhances proliferation and migration	
	T24, SV-HUC-1	**tumor promoter**	tRNA	proliferation ↑, migration ↑	•METTL1 catalyzes the m^7^G tRNA-derived fragment mtiRL	^128^
					•mtiRL binds ANXA2 and enhances Yes1-mediated Tyr24 phosphorylation	
					•promotes nuclear translocation of p-ANXA2-Y24, driving proliferation and migration	
**PCa**	PC3, DU145, 22Rv1	**tumor promoter**	tRNA	growth ↑, proliferation ↑, apoptosis ↓, TIME ↓, immunotherapy resistance ↑	•METTL1 tRNA m^7^G maintains specific tRNA pools and regulates 5′TOGs	^129^
					•loss of 5′TOGs redistributes initiation factors, suppressing translation of growth-promoting genes	
					•activates IFN–STAT1 signaling, modulating the immune microenvironment	
	LNCaP-AI, C4–2	**tumor promoter**	mRNA	proliferation ↑, invasion ↑	•METTL1 installs m^7^G on internal mRNAs such as CDK14	^130^
					•Stabilizes transcripts, promoting castration-resistant progression	
					•p300/SP1 complex upregulates METTL1 transcription	
**ACC**	H295R, SW13	**tumor promoter**	tRNA	growth ↑, proliferation ↑, migration ↑, invasion ↑, glycolysis ↑, TIME ↓, immunotherapy resistance ↑	•METTL1 overexpression (driven by miR-885–5p/CEBPB dysregulation) increases tRNA m^7^G	^131^
					•enhances translation of glycolytic enzyme HK1	
					•suppresses p53 signaling and interferon-mediated antitumor responses	
					•promotes proliferation, migration, invasion, and immune evasion	
**BC**	MCF-7, MDA-MB-231	**tumor suppressor**	tRNA	proliferation ↓, cell cycle arrest (G2/M) ↑	•METTL1/WDR4 catalyze m^7^G modification on 19 specific tRNAs in an enzymatic activity–dependent manner	^91,133^
					•increased m^7^G-modified tRNAs selectively enhance the codon-dependent translation of tumor suppressors GADD45A and RB1	
					**•**GADD45A and RB1 interact with CDKs and CCNB1, enforcing G2/M cell-cycle arrest	
					•METTL1 overexpression potentiates the antitumor efficacy of the CDK4/6 inhibitor abemaciclib in vivo	
	TCGA-BRCA, immune profiling data sets	context-dependent/prognostic	not specified	prognosis ↓, immunosuppression ↑	•METTL1 expression is elevated in invasive BRCA compared with normal tissue, indicating diagnostic relevance	
					•high METTL1 expression correlates with poorer overall and disease-specific survival	
					•METTL1 and coregulated genes positively associate with Tregs and Tfh cells, suggesting a role in shaping an immunosuppressive tumor immune microenvironment	
					•highlights stage-, subtype-, and microenvironment-dependent functional divergence	
**OC**	-	**tumor suppressor**	not studied	TIME ↑	•METTL1 expression inversely correlates with risk score in prognostic models	^133^
					•Associated with increased immune effectors (resting CD4^+^ memory T cells) and reduced M1 macrophages/plasma cells	
					•suggests immunomodulatory role, but direct tumor-intrinsic effects are not experimentally confirmed	
**OS**	HOS, 143B	**tumor promoter**	tRNA	growth ↑, proliferation ↑, migration ↑, apoptosis ↓, chemotherapy resistance ↑	•METTL1/WDR4 tRNA m^7^G modification boosts translation of extracellular matrix genes	^14^
					•enhances mRNA translation of LOXL2, NEK1, and LSM14A	
					•supports proliferation, invasion, and doxorubicin resistance	
	U2OS, 143B	**tumor suppressor**	miRNA	ferroptosis ↑, chemotherapy sensitivity ↑	•METTL1 modifies pri-miR-26a and FTH1 mRNA via m^7^G	^134^
					•REDUCES FTH1 protein, promoting ferroptosis	
					•ncreases chemotherapy sensitivity	
**LS**	AKT-driven zebrafish sarcoma model; human LS cells	**tumor promoter**	tRNA (m^7^G-independent)	protein synthesis ↑, tumor growth ↑	•METTL1 is amplified within the 12q13–15 chromosomal region, frequently amplified in liposarcoma	^135^
					•AKT phosphorylates METTL1 at Ser27, suppressing its methyltransferase activity	
					•catalytically inactive and phosphomimetic METTL1 mutants retain oncogenic activity, demonstrating m^7^G-independent function	
					•METTL1 associates with the multi–tRNA synthetase complex (MSC)	
					•promotes global tRNA aminoacylation, polysome assembly, and protein synthesis, relieving translational bottlenecks under hyperactive AKT signaling	
	METTL1-amplified LS cells	**therapeutic vulnerability**	Ribosome biogenesis–related	drug sensitivity ↑	•METTL1-driven translational amplification creates dependency on ribosome biogenesis	
					•METTL1-amplified liposarcoma cells display heightened sensitivity to low-dose actinomycin D	
					•identifies a nonenzymatic, translation-linked therapeutic vulnerability rather than direct METTL1 inhibition	
**AML**	MOLM-13	**tumor promoter**	tRNA	growth ↑, proliferation ↑	•METTL1 tRNA m^7^G increases Arg-TCT-4–1 tRNA abundance	^82,136^
					•promotes the translation of AGA codon-enriched mRNAs including HMGA2	
					•drives oncogenic proliferation and cytarabine resistance	
**Teratoma**	hiPSCs	**tumor suppressor**	tRNA	proliferation ↑, self-renewal ↑, differentiation ↓, angiogenesis ↓	•METTL1 tRNA m^7^G modification enhances the translation of pluripotency factors OCT4, Nanog, and SOX2	^137^
					•promotes self-renewal and inhibits differentiation	
					•alters angiogenic signaling in teratoma formation	

Glioma provides a clear paradigm of context-dependent METTL1 biology. In IDH–wild-type and high-grade gliomas, METTL1 is strongly upregulated, with expression increasing from low-grade lesions to glioblastoma and correlating with poor survival.^97,98^ In glioma cell models such as U87, METTL1 functions as an oncogene through tRNA m^7^G modification, primarily promoting proliferation. METTL1-mediated tRNA m^7^G accelerates decoding of growth-promoting transcripts and activates mitogenic pathways such as MAPK; METTL1 knockdown reduces ERK1/2 phosphorylation, impairs proliferation, and slows tumor progression. METTL1 also stabilizes metabolic transcripts, notably PGK1 (phosphoglycerate kinase (1), via m^7^G-dependent methylation, thereby enhancing glycolytic flux and supporting the high metabolic demands of glioma. m^7^G regulators, including METTL1, WDR4, and WBSCR22, participate in the wider rewiring of glioma transcriptomes by activating PI3K/AKT/GSK3β, accumulating β-catenin, upregulating Cyclin D1, and enriching cell-cycle, epithelial–mesenchymal transition (EMT), and inflammatory signaling programs.^99,100^ In contrast, in IDH mutant gliomas, METTL1 acquires a tumor-suppressive role by preferentially enhancing translation of genome stability regulators such as GADD45A and RB1. This suppressive role mirrors other tumor contexts in which METTL1 selectively enhances translation of checkpoint regulators rather than oncogenic drivers. In this genetic background, METTL1-driven m^7^G elevates GADD45A and RB1 protein levels, strengthens checkpoint signaling, prolongs G_2_/M arrest, and constrains tumor evolution, while METTL1 loss shortens G_2_/M, compromises genomic integrity, and accelerates tumor progression.^101,102^

METTL1 has been identified as a critical oncogenic regulator in neuroblastoma (NBL), where its expression is markedly upregulated in advanced-stage disease and correlates with poor clinical prognosis.^103^ Functional studies in NBL cell lines (KELLy and BE2C) and xenograft models demonstrate that METTL1 promotes tumor progression by enhancing cell proliferation and migration, whereas its depletion suppresses malignant phenotypes. Genome-wide m^7^G profiling and ribosome nascent-chain complex sequencing revealed that METTL1 knockdown reduces global m^7^G levels and m^7^G-modified tRNA abundance, leading to impaired translation of 339 overlapping genes enriched in oncogenic pathways. Notably, key effectors such as metadherin (MTDH) and programmed cell death 10 (PDCD10) were identified as translational targets dependent on METTL1-mediated tRNA m^7^G modification.^103^ Consistent with these findings, METTL1 silencing significantly decreases translational efficiency, as evidenced by reduced puromycin incorporation, underscoring its essential role in sustaining protein synthesis in NBL cells.^103^

Across head and neck squamous cell carcinoma (HNSCC), ESCC, NPC and oral squamous cell carcinoma (OSCC), METTL1/WDR4 is consistently overexpressed and reinforces PI3K–AKT–mTOR and WNT/EMT pathways through codon-biased translation.^104^ In SCC9, SCC15, KYSE150, and KYSE30 cells, METTL1 functions as an oncogene via tRNA m^7^G modification, promoting proliferation, migration, and invasion while suppressing apoptosis. Particularly, in HNSCC, METTL1/WDR4 enhances translation of EGFR, PIK3CA, and PTEN, collectively driving PI3K–AKT–mTOR signaling and promoting proliferation, EMT, and metastasis.^105^ In ESCC, METTL1 activity causes translational reprogramming and autophagy via selective enhancement of RPTOR translation, sustaining mTORC1 activity and restraining ULK1-driven autophagy; METTL1 suppression reduces RPTOR, attenuates mTORC1, activates ULK1, and shifts cells toward an autophagic, less aggressive state.^104^ In NPC, ARNT-driven overexpression of METTL1/WDR4 supports WNT3A signaling, stabilizes SNAI1, and promotes EMT, proliferation and therapeutic resistance, while METTL1 depletion restores chemosensitivity.^106–108^ In OSCC, METTL1 overexpression promotes resistance to the multitarget TKI anlotinib by enhancing m^7^G tRNA–dependent translational output and driving a metabolic rewiring toward OXPHOS, thereby attenuating antiproliferative and pro-apoptotic responses while fostering a more migratory phenotype. Furthermore, in oral squamous carcinoma, a 5′UTR m^7^G mark on NEK1 stabilizes its mRNA and accelerates G_1_/S transition.^93^

High METTL1 expression is significantly associated with tumor recurrence and aggressive clinical behavior in ameloblastoma (AM). Functional studies demonstrate that METTL1 knockdown markedly suppresses AM cell proliferation, migration, and invasion both in vitro and in vivo, indicating a pro-tumorigenic role for METTL1 in this disease.^109^ Mechanistically, ribosome nascent-chain complex sequencing revealed that METTL1 depletion leads to selective downregulation of translational output from genes enriched in MAPK signaling pathway.^109^ Key downstream effectors, including cyclin D1, matrix metalloproteinase-2 (MMP2), MMP9, and vimentin, exhibit reduced translation upon METTL1 silencing, linking METTL1 activity to cell-cycle progression, extracellular matrix remodeling, and EMT. These findings support a model in which METTL1-driven m^7^G-dependent translational control sustains MAPK pathway activity and promotes the invasive growth of AM.^109^

Papillary thyroid carcinoma (PTC) is a prevalent endocrine malignancy regulated by diverse molecular mechanisms.^110^ Recent evidence highlights a critical oncogenic role for METTL1-mediated tRNA m^7^G modification in PTC initiation and progression, and high expression of METTL1 correlates with poor clinical outcomes. Functionally, METTL1 promotes PTC cell proliferation and metastasis via its tRNA methyltransferase activity, with knockdown impairing growth and migration.^110^ Mechanistically, METTL1 establishes a codon-biased translational program: loss of METTL1 reduces specific m^7^G-modified tRNAs decoding codons enriched in tumor-related mRNAs, notably TNF-α, a proinflammatory and pro-tumorigenic cytokine. METTL1 depletion diminishes TNF-α translation, attenuating NF-κB signaling, whereas exogenous TNF-α partially rescues these effects, validating a “tRNA modification–codon recognition–translation regulation” axis. Tissue microarrays confirm a positive correlation among METTL1, WDR4, and TNF-α, underscoring their cooperative role in driving PTC aggressiveness.^110^

Lung cancer (LC) is one of the most thoroughly characterized METTL1/WDR4-dependent malignancies. Both proteins are upregulated in lung adenocarcinoma and squamous carcinoma, with high expression predicting poor survival. In A549 and NCI-H1299 cells, METTL1 functions predominantly as an oncogene via tRNA m^7^G modification, promoting growth, proliferation, migration, and invasion. Ribosome profiling with nucleotide resolution sequencing (RNC-seq) confirmed that impaired m^7^G tRNA modification upon METTL1/WDR4 depletion decreases translation of mRNAs related to proliferation, invasion, and tumorigenic capacities, while overexpression of the METTL1 downstream target CCND3 partially rescues growth and invasion in METTL1-depleted LC cells. METTL1/WDR4-driven tRNA m^7^G enhances translation of cell-cycle regulators such as CCND3 and CCNE1 and supports AKT–mTORC1 signaling, thereby fostering proliferation, invasion, and xenograft growth.^111^ In EGFR mutant lung adenocarcinoma, METTL1 installs m^7^G on FOXM1 mRNA, increasing its stability and transcriptional activity; elevated FOXM1 suppresses the tumor suppressor PTPN13 and drives resistance to EGFR TKIs. METTL1 depletion reduces FOXM1, restores PTPN13 and reverses gefitinib resistance.^111^ However, in specific contexts METTL1 can exert tumor-suppressive effects through miRNA regulation. In parallel, METTL1/WDR4 can methylate pri-let-7e, resolving a G-quadruplex that impairs processing and thereby increasing levels of tumor-suppressive let-7e, which targets HMGA2, RAS, and MYC. METTL1 inhibits proliferation of LC A549 cells by promoting the processing of pri-let-7e into prelet-7e and mature let-7e, negatively regulating HMGA2, which facilitates EMT and accelerates cancer progression. METTL1 knockdown increases HMGA2 mRNA and protein levels, whereas mature let-7e transfection reverts this upregulation. AKT-mediated phosphorylation of METTL1 reduces this activity, again emphasizing that upstream signaling context dictates whether METTL1 outputs are predominantly oncogenic or partially tumor-restraining.^11^

In GC, METTL1 is frequently overexpressed, correlating with advanced stage, nodal metastasis and poor survival. METTL1 expression is higher in GC tissues (AGS and HGC-27 cells) than in normal tissues, and its regulatory role in promoting proliferation and migration while inhibiting apoptosis may involve tetraspanin 31 (TSPAN31).^112^ Functional studies show that METTL1 knockdown suppresses proliferation, migration, and invasion, implicating an oncogenic role likely mediated through AKT/STAT3-dependent and translational mechanisms analogous to those seen in other gastrointestinal tumors. Moreover, an increasing number of findings suggest that METTL1 contributes to immune evasion by enhancing transcription of immune checkpoints such as PD-1 and CTLA-4 and modulating T-cell and macrophage infiltration.^112,113^

HCC represents one of the clearest examples of METTL1/WDR4-driven oncogenesis. Both proteins are consistently upregulated, and high expression is associated with advanced stage, vascular invasion, poor prognosis, and reduced therapeutic responsiveness. In multiple HCC cell models (Huh7, HepG2, MHCC97H, SNU449), METTL1 functions as a bona fide oncogene acting primarily through tRNA m^7^G modification. METTL1-mediated expansion of the m^7^G-modified tRNA pool preferentially amplifies translation of mRNAs reliant on m^7^G-sensitive codons, including Cyclin A2, EGFR, and VEGFA.^23^ The resulting codon-biased translational rewiring drives cell-cycle progression, activates PI3K–AKT, and MAPK pathways, and promotes EMT and metastatic dissemination. Furthermore, METTL1 increases the growth and migration of HCC cells through the suppression of PTEN signaling.^114^ METTL1 knockdown reduces global m^7^G levels, impairs proliferation and migration, triggers apoptosis, and suppresses HCC growth in xenografts, confirming that METTL1-dependent translation is essential for tumor maintenance. METTL1 also underpins therapeutic resistance. High METTL1 predicts poor response to lenvatinib and radiofrequency ablation (RFA). Under lenvatinib pressure, METTL1-driven tRNA m^7^G enhances EGFR translation, enabling adaptive drug resistance.^94^ Moreover, METTL1 promotes radiotherapy resistance by enhancing DNA double-strand break repair through increased translation of DNA-PKcs and DNA ligase IV, which strengthens nonhomologous end joining (NHEJ).^115^ METTL1 additionally increases translation of NHEJ factors such as DNA-PKcs and DNA ligase IV after ionizing radiation, augmenting DNA double-strand break repair and promoting radioresistance.^116^ Beyond tumor-intrinsic functions, METTL1 remodels the microenvironment through a TGF-β2–PMN-MDSC axis that fosters immunosuppression and post-RFA recurrence. Accordingly, METTL1 overexpression is associated with a reduced TIME and increased infiltration of immunosuppressive PMN-MDSCs in HCC. WDR4 adds a further oncogenic layer via a MYC–WDR4–CCNB1 pathway: c-MYC upregulates WDR4, which stabilizes and promotes translation of CCNB1 by facilitating EIF2A binding, driving G2/M transition, activating PI3K/AKT, and dampening p53-dependent apoptosis, thereby enhancing sorafenib resistance.^117^

ICC exhibits a similarly strong dependency on METTL1. METTL1/WDR4 is markedly overexpressed and correlates with recurrence, vascular invasion, and poor survival.^118^ Functional studies in HuCCT1 and RBE cells demonstrate that METTL1 acts as an oncogene via tRNA m^7^G modification, promoting growth, proliferation, migration, and invasion while suppressing apoptosis. Mechanistic studies show that METTL1-mediated tRNA m^7^G establishes a codon-biased translational landscape that preferentially augments cell-cycle regulators such as Cyclin A2, Cyclin D2, CDK6, and CDK8, as well as EGFR pathway components.^119^ METTL1 depletion reduces m^7^G tRNA levels, induces ribosome stalling and collisions at m^7^G-decoded codons, and increases eIF2α phosphorylation, generating a selective translation-initiation defect that suppresses oncogenic protein synthesis.^119^ Phenotypically, METTL1 knockdown curtails proliferation, migration, and invasion; boosts apoptosis; and abrogates clonogenic growth. Liver-specific genetic ablation demonstrates that METTL1 is required for ICC tumorigenesis in vivo. In addition, METTL1 shapes an immunosuppressive TIME by enhancing chemokines CXCL8/Cxcl5 translation and promoting CXCR2^+^ PMN-MDSCs recruitment, contributing to immunotherapy resistance and establishing an immunosuppressive niche. CXCR2 blockade synergizes with METTL1 inhibition to reduce suppressor cell infiltration and enhance anti-PD-1 efficacy, illustrating how m^7^G-directed translational control modulates immunotherapy responsiveness.^119,120^

In CRC, METTL1 is significantly upregulated and associates with aggressive disease and poor outcome. However, CRC represents a dual-context tumor in which METTL1 can act either as an oncogene or as a tumor suppressor depending on the dominant RNA substrate (tRNA vs miRNA). A study confirmed that METTL1 expression is markedly higher in CRC tissues compared with normal mucosa and positively correlates with poor prognosis. METTL1 promotes CRC progression by enforcing a pro-proliferative translational program and modulating both mRNAs and miRNAs. It accelerates G_1_/S and G_2_/M transitions by suppressing the checkpoint kinase CHEK2, reducing p21 levels and CDC25C phosphorylation, thereby weakening cell-cycle checkpoints. METTL1 knockdown suppresses CRC cell growth and induces G1/S phase arrest, highlighting its role as a cell-cycle regulator.^89^ METTL1 also stabilizes oncogenic transcripts such as RRP9 via m^7^G modification, supporting ribosome biogenesis. In addition, it increases CD155 expression through a PKM-dependent mechanism, facilitating immune evasion via interactions with inhibitory receptors on NK and T cells.^121^ Conversely, in chemotherapy-treated CRC, METTL1 exerts a tumor-suppressive role by enhancing miR-149–3p maturation, reducing S100A4, stabilizing p53, and restoring chemosensitivity. In cisplatin-resistant CRC (CR-CRC), METTL1 expression is reduced; restoration of METTL1 enhances chemosensitivity through activation of a miR-149–3p/S100A4/p53 axis. In this pathway, m^7^G-dependent processing of miR-149–3p represses S100A4, stabilizes p53, and promotes apoptosis. Overexpression of either METTL1 or miR-149–3p increases p53 protein levels and amplifies the cytotoxic effects of cisplatin, which can be reversed by upregulating S100A4.^122^ Interestingly, METTL1 can also act in a tumor-restraining manner in CRC by promoting m^7^G-dependent maturation of let-7e, leading to HMGA2 repression and inhibition of proliferation and invasion.^123^ These context-dependent outputs illustrate how METTL1 can either support or oppose malignant behavior depending on the dominant downstream circuitry.^122,123^

BCa displays one of the most prominent dependencies on METTL1 among solid tumors. METTL1 is markedly overexpressed in urothelial carcinoma and correlates with high grade, muscle invasion, metastasis, and reduced survival, whereas WDR4 is not uniformly elevated, suggesting that catalytic overactivation is largely METTL1-driven.^124–126^ Across T24, UMUC-3, and SV-HUC-1 cells, METTL1 consistently functions as an oncogene via both tRNA and miRNA m^7^G pathways. METTL1-mediated m^7^G tRNA modification drives BCa cell progression by preferentially enhancing the codon-biased translation of oncogenic transcripts, including EGFR and the extracellular matrix protein EFEMP1 (EGF-containing fibulin-like extracellular matrix protein 1), thereby amplifying EGFR ligand-dependent autophosphorylation and sustaining downstream PI3K–AKT and MAPK signaling cascades that collectively promote tumor cell proliferation, survival, migration, and invasion.^124^ METTL1 silencing diminishes global translation, induces G_1_ arrest, suppresses EMT, and dramatically reduces tumor growth and metastasis in xenograft and zebrafish models. Furthermore, Xie et al. reported that METTL1 drives BCa progression by promoting m^7^G-dependent processing of pri-miR-760 (via miRNA-seq and MeRIP-seq), leading to ATF3 (activating transcription factor 3) degradation and promoting expression of TROP2 via tRNA-mediated translational enhancement, adding further oncogenic circuits relevant to bladder cancer aggressiveness.^124–126^ ATF3 overexpression significantly represses BCa cell proliferation and migration by regulating cell cycle and EMT-associated proteins, revealing a METTL1/miR-760/ATF3 regulatory axis as a potential therapeutic target.^127^ Surprisingly, emerging evidence indicates that m^7^G-3′-tiRNA Lys­(TTT) (mtiRL), a novel m^7^G-modified tRNA-derived small RNA catalyzed by METTL1, plays an oncogenic role in BCa in vitro and in vivo. Specifically, METTL1 catalyzes the formation of mtiRL, which selectively binds to the oncoprotein annexin A2 (ANXA2). This binding promotes Tyr24 phosphorylation of ANXA2 by enhancing its interaction with Yes proto-oncogene 1 (Yes1), facilitating nuclear translocation of phosphorylated ANXA2 (p-ANXA2-Y24) in BCa cells and thereby augmenting proliferation and migration.^128^

In prostate cancer (PCa), especially advanced and castration-resistant disease (CRPC), METTL1 is significantly upregulated, with high expression predicting poor survival.^129^ In PC3, DU145, 22Rv1, LNCaP-AI and C4–2 cells, METTL1 acts as an oncogene via both tRNA and mRNA m^7^G pathways. Transcriptional activation by a p300/SP1 complex enhances METTL1 expression, and its catalytic activity installs internal m^7^G on mRNAs such as CDK14, stabilizing them and promoting castration-resistant progression. Genetic deletion of METTL1 reduces CDK14, suppresses proliferation, and slows tumor growth in vitro and in vivo.^130^ In PTEN-deficient mouse models, METTL1 loss profoundly suppresses tumorigenesis and remodels the immune microenvironment, increasing M1-like macrophages and CD8^+^ T-cell infiltration while diminishing immunosuppressive myeloid subsets. At the tRNA level, METTL1 protects specific tRNAs and regulates the biogenesis of 5′ tRNA-derived fragments (5′tRFs or 5′TOGs), which reprogram translation during oncogenic stress. In METTL1-deficient prostate cancer cells, loss of 5′TOGs redistributes initiation factors away from capped mRNAs, suppressing translation of growth-promoting and stress-adaptive gene sets and activating IFN-STAT1 signaling, which in turn promotes recruitment of cytotoxic immune populations and converts the prostate TIME into a more tumoricidal state.^38,129^

In adrenocortical carcinoma (ACC), METTL1 is aberrantly overexpressed and acts as a central oncogenic regulator that integrates m^7^G-dependent translational control with tumor growth, proliferation, migration, invasion, metabolic rewiring, and immune evasion.^131^ Functionally, elevated METTL1 enhances ACC cell proliferation, migration, and invasion, whereas its genetic silencing markedly suppresses tumor growth in xenograft models. At the mechanistic level, METTL1 promotes a glycolytic phenotype by upregulating the rate-limiting enzyme hexokinase 1 (HK1), linking m^7^G-driven translation to metabolic reprogramming, with miR-885–5p and CEBPB identified as potential upstream regulators of METTL1 expression. Mechanistically, dysregulation of a miR-885–5p/CEBPB–METTL1 axis in ACC drives METTL1 overexpression, which enhances m^7^G-dependent translation of glycolytic targets such as HK1, promoting metabolic reprogramming, attenuation of p53 signaling, and suppression of interferon-mediated antitumor immunity, thereby coupling epitranscriptomic control to tumor progression and immune escape. In parallel, high METTL1 expression is associated with an immunosuppressive tumor microenvironment, characterized by reduced CD8^+^ T-cell infiltration and increased macrophage abundance. Clinically, METTL1 is a key component of an m^7^G-based prognostic signature that robustly stratifies ACC patients, correlates with suppressed antitumor immune responses, and predicts altered sensitivity to immune checkpoint blockade and mitotane therapy, underscoring its dual role as a driver of malignancy and a determinant of therapeutic vulnerability in ACC.^131^

Emerging evidence indicates that METTL1-mediated tRNA m^7^G modification plays a critical role in breast cancer (BC) by regulating cell proliferation, cell cycle progression, and therapeutic response. Notably, comprehensive analyses using m^7^G tRNA MeRIP-seq and ribosome profiling (Ribo-seq) have demonstrated that METTL1-mediated tRNA m^7^G modification exerts inhibitory effects on BC cell cycle progression and proliferative capacity, supporting a tumor-suppressive function in this tumor context. In BC, METTL1 and its cofactor WDR4 are downregulated at both mRNA and protein levels, contrasting with their oncogenic roles in other cancers. This downregulation further supports the concept that METTL1 plays a context-dependent role and functions predominantly as a tumor suppressor in BC. Functionally, METTL1 acts in an enzymatic activity-dependent manner, increasing m^7^G levels on 19 specific tRNAs to selectively enhance codon-dependent translation of tumor suppressors GADD45A and RB1, inducing G2/M cell cycle arrest and inhibiting proliferation.^132^ Mechanistically, enhanced translation of GADD45A and RB1 promotes their interaction with cyclin-dependent kinases (CDKs) and cyclin B1 (CCNB1), thereby enforcing G2/M phase cell cycle arrest and restraining breast cancer cell growth.^91^ In vivo, METTL1 overexpression augments the antitumor efficacy of the CDK4/6 inhibitor abemaciclib.^91^ Bioinformatic analyses reveal that METTL1 expression is significantly higher in invasive BC tissues compared with normal controls, demonstrating diagnostic potential. Survival analyses indicate that patients with low METTL1 expression have superior five-year overall and disease-specific survival, whereas elevated METTL1 correlates with poor prognosis. This apparent paradox underscores the complex and context-dependent role of METTL1 in breast cancer biology, potentially influenced by tumor stage, molecular subtype, or microenvironmental factors. Additionally, METTL1 and associated core genes positively correlate with immunosuppressive populations, including regulatory T cells (Tregs) and follicular helper T cells (Tfh), implicating METTL1 in shaping the TIME.^38,132^

METTL1 has emerged as a clinically relevant RNA modification–associated regulatory factor in ovarian cancer (OC), where its aberrant expression is strongly linked to unfavorable patient outcomes.^133^ As a differentially expressed epitranscriptomic regulator, METTL1 was incorporated into a prognostic signature constructed using least absolute shrinkage and selection operator (LASSO) regression analysis, in which the derived risk score showed an inverse correlation with METTL1 expression levels. Moreover, the risk score was positively associated with resting CD4^+^ memory T cells, while exhibiting negative correlations with M1 macrophages and plasma cells, highlighting a potential role for METTL1 in modulating the immune microenvironment of OC.^133^ This signature functions as an independent prognostic model for patient stratification, outcome prediction, and evaluation of responsiveness to immunotherapeutic strategies. Despite the important role played by METTL1 in the pathological features and prognostic framework of OC, the study did not provide direct experimental evidence establishing a causal link between METTL1 and malignant cellular behaviors in OC.

Osteosarcoma (OS) is likewise characterized by robust METTL1/WDR4 upregulation and strong dependence on tRNA m^7^G. Enhanced m^7^G-modified tRNA pools support high translational output, proliferation, invasion, and chemoresistance,^14^ while METTL1 knockdown reduces m^7^G levels, induces cell-cycle arrest and suppresses tumor growth.^14^ METTL1/WDR4 activity preferentially boosts translation of extracellular matrix–remodeling genes, facilitating invasion and doxorubicin resistance.^14^ At the mRNA level, m^7^G on NEK1 and LSM14A increases their stability and translation, contributing to cell-cycle progression and RNA granule remodeling. METTL1 stimulates OS malignancy by augmenting mRNA translation of lysyl oxidase-like 2 (LOXL2), a key downstream target required for its function in osteosarcoma.^14^ On the other hand, METTL1 also modulates ferroptosis sensitivity through m^7^G-dependent regulation of FTH1 and pri-miR-26a, linking tRNA/mRNA methylation to oxidative stress and therapeutic vulnerability.^134^ Interestingly, METTL1 reduces FTH1 protein levels by targeting pri-miR-26a and FTH1, evoking ferroptosis and promoting osteosarcoma cell sensitivity to chemotherapy.^38^

Amplification of the 12q13–15 chromosomal region is a recurrent event in human cancers, particularly in liposarcoma (LS), where it drives coordinated overexpression of multiple oncogenes.^135^ Using an AKT-driven zebrafish model of sarcomagenesis, METTL1 was identified as an unexpected cooperating oncogene within this amplicon. Notably, although METTL1 is best known as a tRNA m^7^G methyltransferase, its oncogenic activity in LS was shown to be independent of its catalytic function. AKT-mediated phosphorylation of METTL1 at Ser27 inhibits its methyltransferase activity, yet phosphomimetic and catalytically inactive METTL1 mutants retained full oncogenic potential.^135^ Mechanistically, METTL1 associates with the multi–tRNA synthetase complex (MSC), promoting global tRNA aminoacylation, polysome formation, and protein synthesis independently of m^7^G deposition. In the context of hyperactive AKT signaling, METTL1 overexpression alleviates aminoacylation-dependent constraints on translation, thereby maximizing protein synthesis and tumor cell growth. Importantly, METTL1-amplified LS cells display heightened sensitivity to low-dose actinomycin D, revealing a therapeutic vulnerability linked to ribosome biogenesis rather than enzymatic inhibition of METTL1 itself.^135^

In acute myeloid leukemia (AML), both METTL1 and WDR4 are markedly upregulated, and elevated expression associates with aggressive disease and poor survival. In MOLM-13 cells, METTL1 functions as a potent oncogene through tRNA m^7^G modification. METTL1 increases the abundance of m^7^G-modified tRNAs, particularly Arg-TCT-4–1, and promotes translation of mRNAs enriched in the corresponding AGA codon, including cell cycle regulators (such as HMGA2), thereby driving oncogenic transformation and proliferation.^82^ Furthermore, METTL1 knockout effectively inhibited leukemic stem cells growth, and METTL1 knockout mice displayed longer survival than wild-type controls.^136^ Overexpression of Arg-TCT-4–1 alone can phenocopy METTL1-driven transformation, indicating that the dominant oncogenic output in AML is tRNA-centric.^82^ METTL1 loss reduces global m^7^G, destabilizes modified tRNAs, and increases tRNA-derived small RNA (tsRNA) production. This weakens translational efficiency, causes G_1_ arrest, caspase-3 activation, BCL2 downregulation, apoptosis, and loss of leukemia-initiating capacity. Nascent proteome profiling shows that either METTL1 knockdown or transfection of total tRNA derived from METTL1-deficient AML cells decreases overall translation efficiency, confirming the critical role of METTL1 in protein synthesis control. METTL1 overexpression confers cytarabine resistance by maintaining translational buffering under genotoxic stress, whereas METTL1 silencing sensitizes AML cells to cytarabine.^136^

Other contexts underscore the versatility of METTL1/WDR4 biology. In human-induced pluripotent stem cells, altered m^7^G tRNA methylation affects teratoma formation, in part by modulating translation of stemness factors such as OCT4, Nanog, and SOX2 and influencing self-renewal and differentiation programs.^1,137,138^

More broadly, WDR4 itself is emerging as an independent oncogenic driver. As the WD-repeat scaffold of the METTL1 complex, WDR4 is required for METTL1 stability, proper tRNA positioning, and generation of the tRNA m^7^G methylome. Across cancers, WDR4 overexpression correlates with advanced stage and poor outcome, and its depletion phenocopies METTL1 loss by reducing tRNA m^7^G and limiting growth. In addition, WDR4 can operate independently of METTL1, for example in HCC-associated tumor-associated macrophages, where it partners with eIF4E2 to promote selective translation of ABCA1, sustaining an immunosuppressive macrophage phenotype.^139^

Taken together, these data depict METTL1/WDR4 as highly context-dependent regulators. In most malignancies, —including HCC, ICC, CRC, gastric, esophageal, nasopharyngeal and head and neck carcinomas, lung and bladder cancers, prostate cancer, AML, glioma and osteosarcoma—they act as classical oncogenes by expanding m^7^G-modified tRNA pools, relieving ribosome pausing at specific codons and selectively increasing the translation of codon-enriched oncogenic transcripts controlling proliferation, survival, metabolism, DNA repair, and immune escape. High METTL1/WDR4 expression in these contexts consistently marks aggressive disease and poor therapy response. In contrast, in IDH mutant gliomas, BRCA1-deficient breast cancers, osteosarcoma and ovarian cancer, METTL1/WDR4-dependent m^7^G preferentially supports translation of genome stability and checkpoint regulators such as GADD45A and RB1, thereby maintaining genomic integrity and constraining tumor progression.

This dualistic behavior has major implications for drug discovery. While targeting METTL1/WDR4 is an attractive strategy in tumors where their activity clearly drives malignancy and therapy resistance, systemic inhibition may be harmful in settings where METTL1/WDR4 preserves genome stability or where normal tissues, particularly hematopoietic, cardiac and neural compartments, are highly dependent on m^7^G homeostasis. A nuanced understanding of codon usage patterns, dominant transcriptomes and signaling states in each tumor type will therefore be essential to define the therapeutic window and to rationally design small-molecule inhibitors, degraders or context-selective modulators of the METTL1/WDR4 axis for clinical applications.

### METTL1/WDR4 Inhibitors

From a therapeutic perspective, METTL1/WDR4 represents an attractive but complex target. As METTL1 belongs to the SAM-dependent class I methyltransferase family, its catalytic pocket shares structural features with other methyltransferases, raising issues of selectivity. However, the unique partnership with WDR4, the composite tRNA-binding surface, and the dynamic regulatory *N*-terminal region of METTL1 provide additional opportunities for allosteric or interface-targeting strategies. The fact that METTL1/WDR4 mainly drives selective translational rewiring, rather than globally shutting down protein synthesis, suggests that partial inhibition might preferentially attenuate oncogenic programs while sparing normal tissues that are less dependent on these codon-biased translational circuits. At the same time, the involvement of METTL1/WDR4 in stress adaptation and miRNA regulation argues that inhibitor development must consider possible context-dependent effects on immune responsiveness and normal tissue homeostasis, particularly how these effects might influence the efficacy and safety of potential therapies in cancer treatment.

Early efforts to pharmacologically target METTL1 have naturally focused on SAM-competitive inhibitors, given that METTL1 is a classical *S*-adenosyl- *l* -methionine–dependent methyltransferase. Among these, *S* **-adenosyl-** l **-homocysteine** (**SAH**) and the natural nucleoside analog **sinefungin** represent the most informative chemical tools currently available (Figure 5A). SAH, the physiological reaction product of methyl transfer, binds the METTL1 cosubstrate pocket with low-micromolar affinity and acts as a potent feedback inhibitor. Although its IC_50_ cannot be directly measured in standard luminescence-based assays due to assay interference, thermal shift experiments indicate that SAH stabilizes METTL1 and the METTL1/WDR4 complex to a degree comparable to sinefungin, supporting a low-micromolar binding potency. Structurally, SAH engages a conserved network of hydrogen bonds within the SAM-binding pocket, interacting with residues such as E107, N140, A141, and D163, thereby defining the canonical anchoring interactions required for methyl donor recognition (Figure 5B).^140^

**Figure 5:**
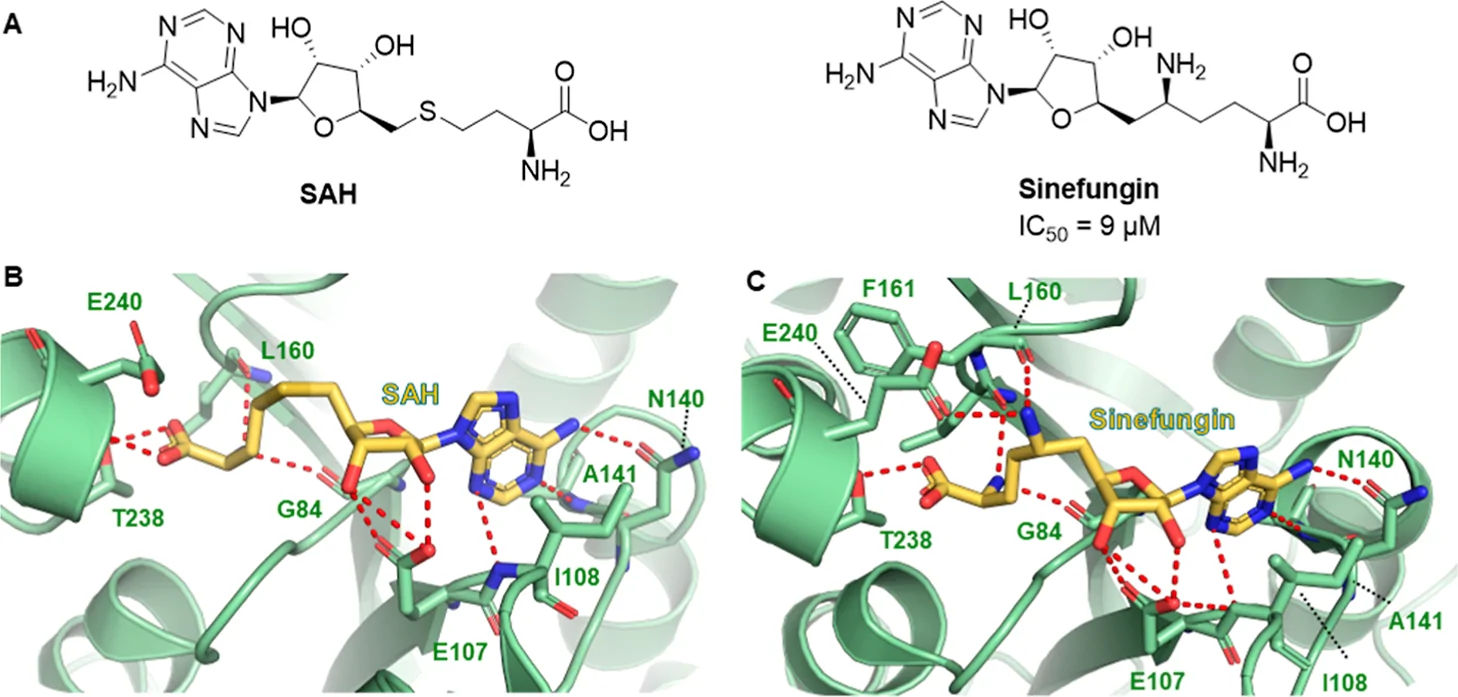
(A) Chemical structures of **SAH** and **sinefungin**. (B,C) Crystal structures of METTL1 bound to (B) **SAH** (PDB ID: 7OGJ) and (C) **sinefungin** (PDB ID: 7PL1). METTL1 is shown as a green cartoon, with key residues involved in polar contacts being shown as sticks. SAH and sinefungin are shown as yellow sticks. Hydrogen bonds are depicted as red dotted lines.

**Sinefungin** (Figure 5A), a natural SAM analog in which the sulfonium methyl group is replaced by an amino methyl group, is currently one of the best-characterized small-molecule inhibitors of METTL1. Using a luminescence-based enzymatic assay with the full METTL1/WDR4 complex and a pri-let-7e–derived RNA substrate, sinefungin inhibits METTL1 activity with an IC_50_ of approximately 9 μM. Importantly, sinefungin has enabled the first high-resolution structural characterization of a METTL1–inhibitor complex. X-ray crystallography at 1.85 Å resolution reveals that sinefungin occupies the SAM cosubstrate pocket in a pose closely overlapping that of SAH, while forming additional stabilizing interactions through its primary amine, including a salt bridge with E240 (Figure 5C). These structural data confirm that sinefungin acts as a direct competitive inhibitor of SAM binding and provide an essential framework for structure-guided inhibitor development.^140^

Despite their value as mechanistic probes, both **SAH** and **sinefungin** suffer from intrinsic limitations as drug candidates, including high polarity, poor cell permeability, and lack of selectivity across the broader methyltransferase family. Nevertheless, **sinefungin** has demonstrated proof-of-concept biological activity in disease models. In particular, pharmacological inhibition of METTL1 with **sinefungin** reduces m^7^G deposition on target RNAs and mitigates tissue damage in myocardial ischemia–reperfusion injury, supporting the therapeutic relevance of METTL1 inhibition in vivo. Collectively, **SAH** and **sinefungin** define the pharmacological starting point for METTL1 targeting and establish the SAM-binding pocket as a tractable site for the development of more selective, drug-like METTL1 inhibitors.^140^

In a recent study, Nai et al. reported a diverse set of fragment-like ligands identified through structure-based docking and biochemical screening, several of which inhibit the METTL1/WDR4 complex with micromolar potency (Figure 6). Among them, compounds **1a**, **1b**, and **1c** provide the first chemical foothold for developing selective inhibitors of METTL1 and illustrate how the SAM-binding pocket can be engaged by non-nucleotide, low-molecular-weight chemotypes.^140^ Compounds **1a** and **1b** (Figure 6) were derived from high-throughput docking of nearly 5000 adenine derivatives positioned to exploit the adenine-recognition region of the SAM-binding pocket. Both compounds inhibit METTL1 with IC_50_ values of 144 μM and 187 μM, respectively, while achieving ligand efficiencies (0.34 and 0.31 kcal·mol^–1^ per heavy atom) that are unusually high for early methyltransferase inhibitors. Despite their structural simplicity, each compound recapitulates key SAM-like interactions. Compound **1b** shows promising selectivity against the m^6^A-RNA methyltransferases METTL3/14 and METTL16. MD simulations demonstrated that the adenine cores of both compounds remain tightly anchored in the SAM subpocket, retaining an RMSD < 4 Å from the SAH-bound reference structure across the majority of trajectories. Compound **1a** forms a well-defined hydrogen-bonding network with backbone atoms of S139 and I83, while its dihydroxyethyl thioether group establishes additional polar contacts with P29 and D163 and occupies a hydrophobic groove delineated by P162 and I83. Compound **1b** maintains analogous interactions through its adenine ring but distinguishes itself through a protonated piperazine substituent that forms a persistent salt bridge with D163, a residue repeatedly implicated as a key anchoring point for SAM-competitive ligands. Notably, compound **1b** exhibits selectivity for METTL1 over METTL3/14 and METTL16, an attribute uncommon among SAM mimics and highly desirable for future optimization.^140^ Compound **1c** (Figure 6), an adenosine mimic identified during the biochemical screening of a curated library, expands the chemical space beyond adenine derivatives while retaining a high ligand efficiency (0.31 kcal·mol^–1^ per heavy atom). Although less potent (IC_50_ = 277 μM), compound **1c** displayed striking stereoselectivity in MD simulations. Only the (*S,S*) enantiomer achieved a stable SAM-competitive pose, maintaining low RMSD values across 5 of the 8 simulation replicas, whereas the (*R,R*) enantiomer exhibited unstable and poorly defined binding. The productive enantiomer places its adenine moiety in the canonical SAM position, sustaining polar contacts with S139 and A141, and orients its hydroxylated pyrrolidine ring toward D163 and S27 to form a stabilizing combination of salt-bridge and hydrogen-bond interactions. This stereochemical preference strongly suggests that isolation or synthesis of the (*S,S*) enantiomer would lead to substantially enhanced potency and highlights the importance of optimized 3D topology for engaging the METTL1 pocket.^140^

**Figure 6:**
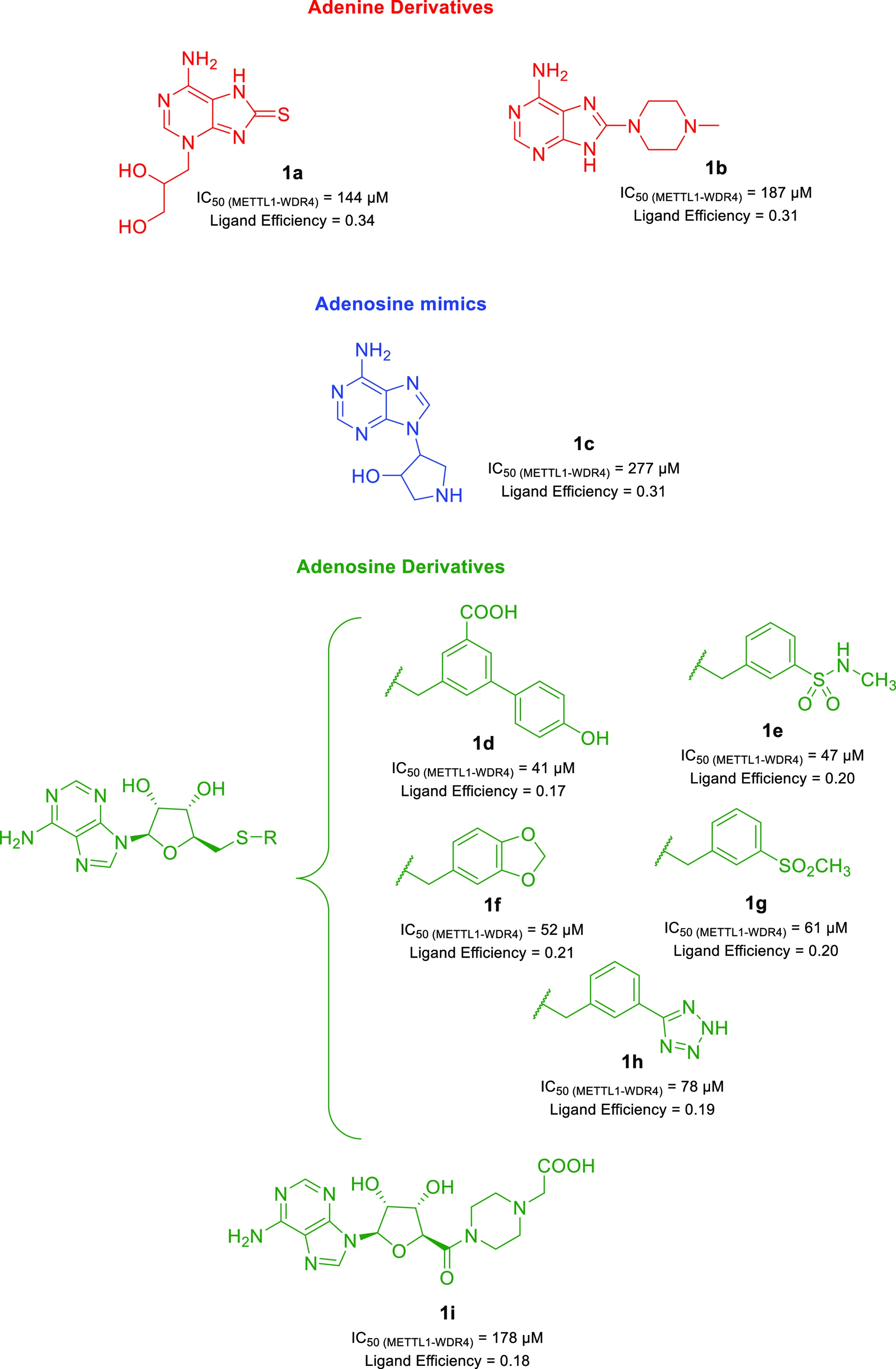
Chemical structures of fragment-like inhibitors identified against the METTL1/WDR4 complex (**1a–i**). Representative METTL1 ligands identified through structure-based docking and biochemical screening are grouped according to chemotype: adenine derivatives (compounds **1a–b**, red), adenosine mimics (compound **1c**, blue), and adenosine-derived inhibitors (compounds **1d–i**, green). For each compound, the corresponding IC_50_ values against the METTL1/WDR4 complex and ligand efficiency (LE) are reported. These fragment-sized chemotypes engage the SAM-binding pocket through conserved adenine-recognition interactions while exploring distinct substituent vectors, highlighting the druggability of the METTL1 active site and providing early chemical starting points for the development of selective m^7^G writer inhibitors.

Among the adenosine-derived inhibitors found by the Caflisch’s team, 26 molecules were selected from a broader panel of SARS-CoV-2 mRNA cap methyltransferase binders. These compounds were generated by bioisosterically replacing the methionine subunit of SAM, and many incorporate a negatively charged group designed to mimic the methionine carboxylate. Five derivatives (**1d**–**1h**) exhibited METTL1 inhibition with IC_50_ values < 100 μM, while compound **1i** exhibited an IC_50_ value < 200 μM (Figure 6), and the most potent member of the series, compound **1e**, stabilized both METTL1 and the METTL1/WDR4 complex in thermal shift assays. Although these molecules interact with METTL1, they generally feature a high number of heavy atoms and thus suffer from low ligand efficiency. Moreover, their close structural similarity to SAM leads to measurable off-target binding to other human methyltransferases, such as glycine-*N*-methyltransferase. Nevertheless, compounds **1d, 1e**, **1g**, and **1i** displayed notable selectivity over METTL3/14 and moderate selectivity over METTL16. To further characterize the binding, compound **1d** was subjected to eight independent 200 ns molecular dynamics (MD) simulations. The simulations were initialized by aligning the adenine ring of the inhibitor to that of **SAH** in the METTL1 crystal structure (PDB ID: 7OGJ). Across the ensemble of runs, compound **1d** maintained a stable binding pose, with the adenine ring deviating by < 4 Å from its crystallographic reference in seven of the eight trajectories. Consistent with this, the adenosyl portion of compound **1d** was predicted to adopt a conformation nearly identical to that of SAH and to engage the same anchoring interactions within the SAM-binding pocket. The substituted biphenyl group extends toward the solvent-exposed region; the phenolic OH forms a hydrogen bond with the backbone NH of M30, while the benzoate moiety establishes a salt bridge with the side chain amine of K18 and additional electrostatic contacts with R109. Together, these simulations support a stable and well-defined binding mode for compound **1d**, combining canonical adenosyl recognition with distinct substituent–driven interactions at the periphery of the active site.^140^

Taken together, the structural, biochemical, and computational analyses converge to demonstrate that METTL1 is amenable to be inhibited by compact, non-nucleotide chemotypes. The fact that compounds **1a**, **1b**, and **1c** can faithfully recapitulate core SAM interactions despite their minimalist scaffolds underscores the druggability of the METTL1 adenine subsite. Moreover, the consistent involvement of residues S139, A141, D163, P162, and I83 across multiple binding modes establishes a clear interaction blueprint for structure-based optimization. Although these early inhibitors are not sufficiently potent to displace SAH for cocrystallization, the extensive MD simulations provide atomistic models that can guide fragment-growing, functionalization of pocket-facing vectors, and stereochemically informed ligand design. Collectively, these compounds represent the first credible chemical starting points for selective modulation of METTL1 activity and pave the way for the development of high-affinity m^7^G writer inhibitors with therapeutic and chemical biology applications.^140^

More recently, Hou et al. identified a putative METTL1/WDR4 inhibitor through a hybrid virtual screening of the ChemDiv library followed by phenotypic screening.^141^ Among the 19 molecules found, **SA91–0178 (2)** (Figure 7) emerged as the most effective compound, showing robust anti-inflammatory activity in LPS-stimulated macrophages without detectable cytotoxicity at concentrations <5 μM. Molecular docking suggested tight binding of **2** to METTL1, which was validated experimentally by SPR (*K*_D_ = 4.066 μM) and CETSA. Moreover, mutating the predicted binding residues abolished compound–protein interaction, confirming the specificity of binding. Functionally, **2** suppressed LPS-induced m^7^G RNA modification in RAW264.7 cells, human PBMC-derived macrophages, and mouse BMDMs; and reduced the production of TNF-α and IL-1β. These results suggest **2** as a pharmacological inhibitor of METTL1, although the authors did not evaluate the in vitro inhibition of methyltransferase activity. In vivo evaluation demonstrated that intraperitoneal administration of **2** (20 mg/kg) was well tolerated, with no alterations in serum biochemical parameters, tissue histology, or macrophage homeostasis.^141^ Pretreatment with **2** at various doses ameliorated kidney injury in both CLP-induced sepsis and renal ischemia-reperfusion (I/R) models, lowering serum creatinine and BUN levels and reducing histopathological damage. The treatment also decreased renal macrophage infiltration and repressed SARM1 protein and mRNA expression in F4/80^+^ macrophages. Similar protective effects, including reduced macrophage accumulation and diminished Sarm1 expression, were observed across liver, lung, and heart tissues. Compound **2** likewise conferred renoprotection in I/R-induced AKI, decreasing tissue injury, macrophage infiltration, and SARM1 levels. Importantly, the compound failed to provide additional benefit in macrophage-specific *Mettl1*-deficient mice, demonstrating that its anti-inflammatory and organ-protective effects depend on METTL1 in macrophages. Therapeutically relevant postinjury administration (24 h after CLP or I/R) also mitigated systemic and tissue inflammation. Although macrophage recruitment was only modestly reduced, **2** markedly suppressed proinflammatory gene expression, including Tnf, in renal F4/80^+^ macrophages and improved multiorgan injury.^141^

**Figure 7:**
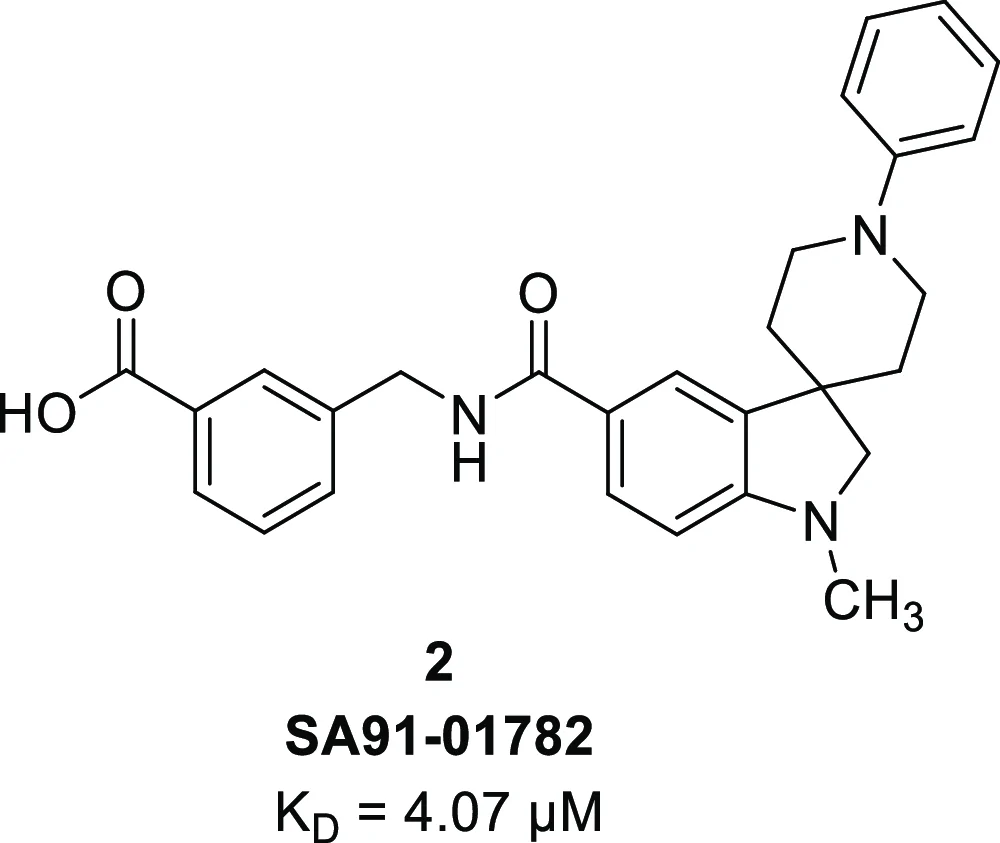
Structure of compound **2**.

The recent patent from Storm Therapeutics WO2025/104443 reported the first comprehensive and pharmacologically advanced class of small-molecule inhibitors targeting the METTL1/WDR4 complex, establishing a structurally coherent and high-affinity chemical framework for modulating tRNA m^7^G methylation.^146^ Within this extensive series, compounds **3a**–**g** emerge as representative and highly informative exemplars, collectively defining the optimal structure–activity relationship (SAR) features required for potent METTL1 inhibition (Figure 8 and Table 2).

**Figure 8:**
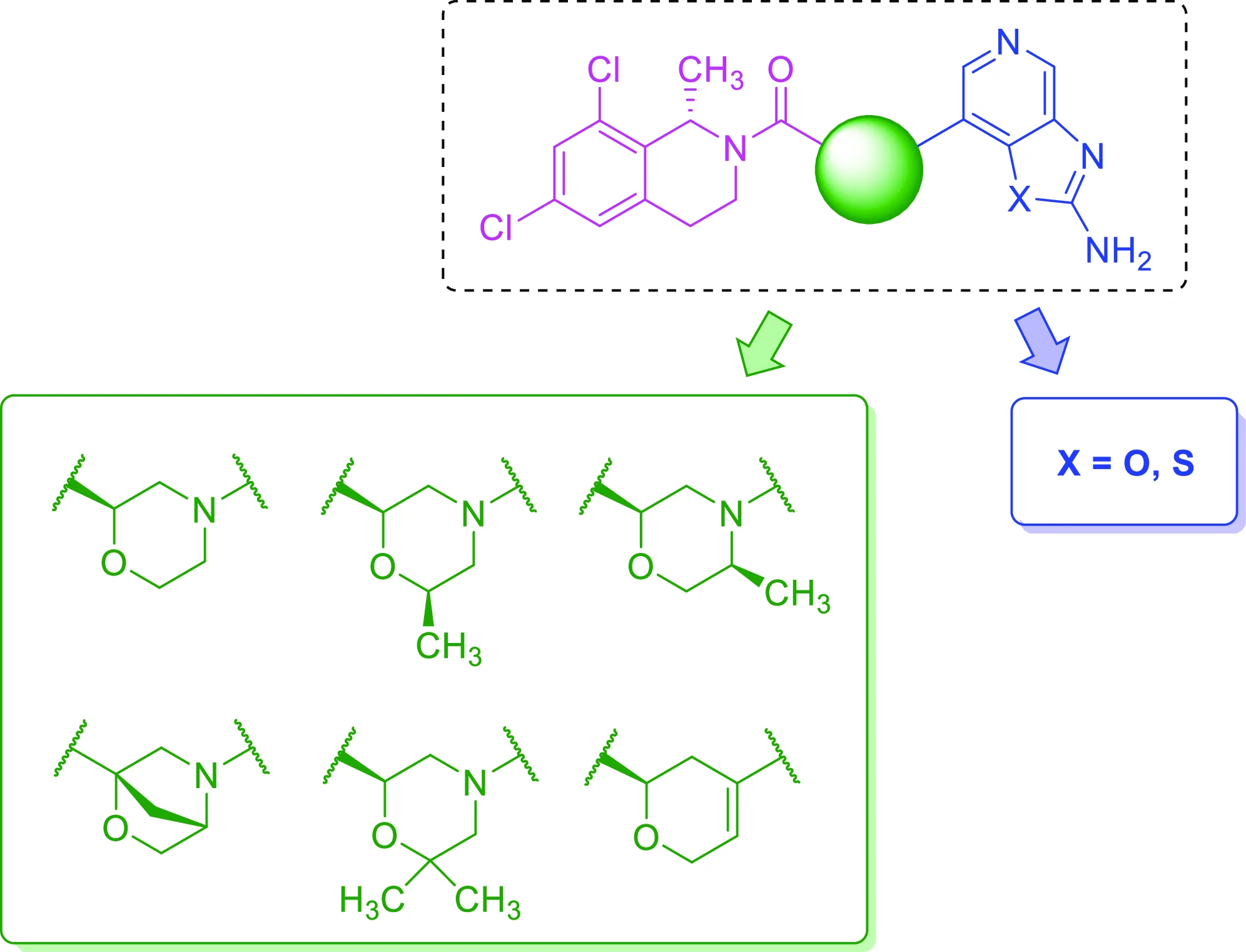
General structure for compounds **3a**–**g**.

**Table 2:**
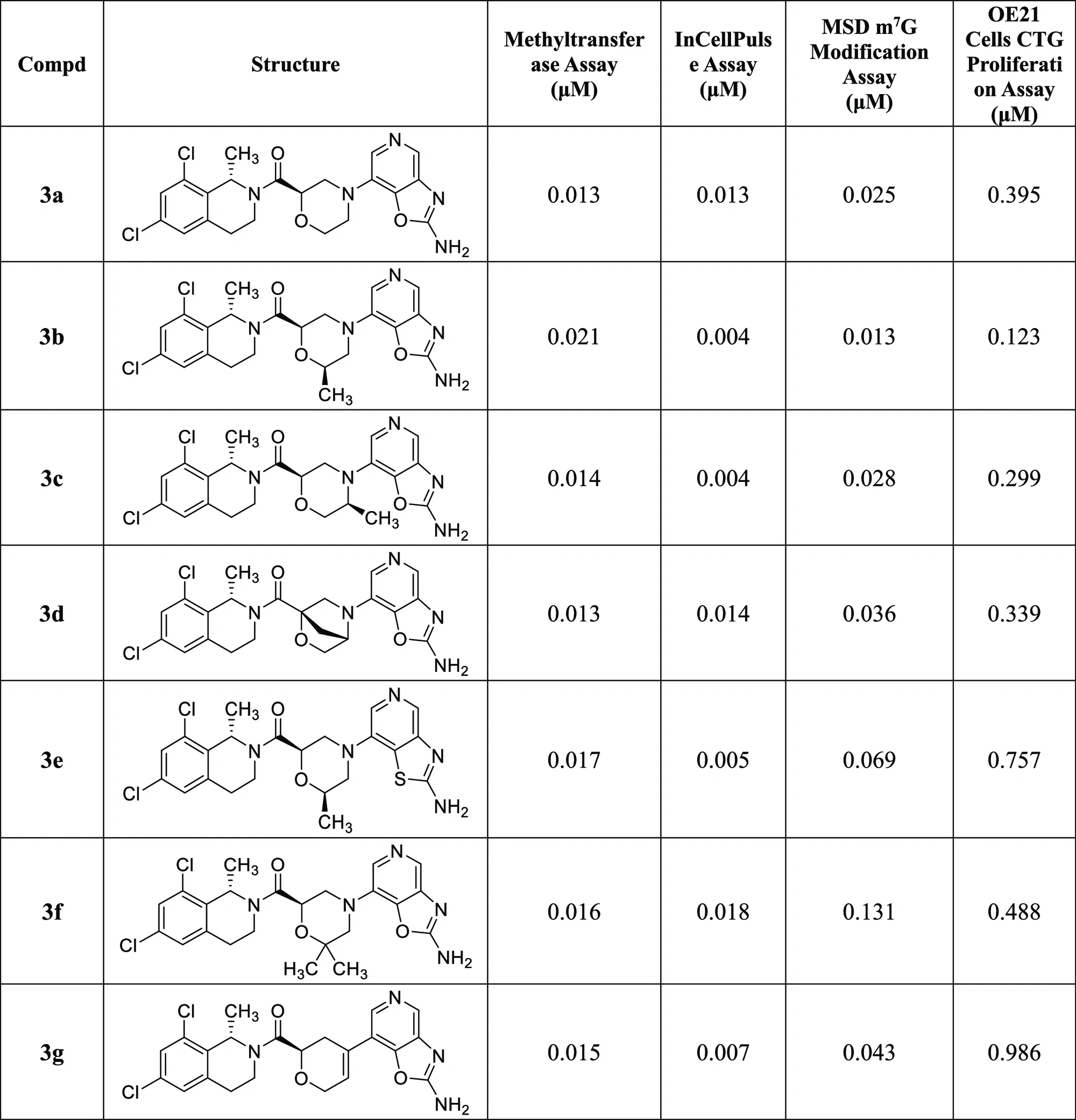
Structures and Biochemical/Biological Activity Profiles of Compounds **3a–3g**

These analogues share a common 1,2,3,4-tetrahydroisoquinoline (THIQ) core bearing a 1-methyl substituent, typically administered as racemates or enriched in the preferred *S*-enantiomer. Potency within this subset is critically dependent on the presence of electron-withdrawing groups at the 6- and 8-positions of the isoquinoline ring, a feature consistently retained across compounds **3a**–**3g** (in fact, they present two chlorine atoms at positions 6 and 8) (Figure 8 and Table 2). Such substitutions are thought to optimize anisotropic electronic distribution within the THIQ scaffold, thereby enhancing productive interactions with the METTL1 catalytic environment, including the SAM-binding region. The THIQ nitrogen is linked via an amide bond to a conformationally constrained six-membered saturated ring, which in turn connects to a bicyclic 6 + 5 heteroaromatic system bearing an essential 2-amino substituent. This amino group is conserved in all highlighted compounds and is indispensable for activity, likely mediating key directional hydrogen bonds within the RNA-recognition surface of METTL1.^146^ Based on the biochemical data, a coherent SAR profile can be delineated. The THIQ core is essential for activity, and potency is highly dependent on the presence of electron-withdrawing substituents at the 6- and 8-positions. Removal of an electron-withdrawing group at the 8-position results in a clear loss of potency, underscoring the importance of the electronic environment of the bicyclic system. The six-membered aliphatic ring linking the THIQ amide is broadly tolerated, including small alkyl substituents adjacent to heteroatoms, methylene bridges, or local unsaturations. In contrast, expansion to seven-membered rings or conversion to spirocyclic bicyclic systems leads to a marked reduction in activity. A five-membered pyrrolidine ring can replace the six-membered morpholine core without significant loss of potency, and functionalization at the carbon adjacent to the carbonyl with methyl, methoxy, or fluorine substituents is also well tolerated. With respect to the bicyclic heteroaromatic moiety, removal of the embedded five-membered ring and retention of only the pyridine core causes a dramatic decrease in potency, indicating that the fused 6 + 5 ring system contributes critical interactions within the METTL1/WDR4 binding site. The 2-amino substituent on this heterobicycle is similarly indispensable, as its deletion substantially reduces activity. When this amino group is functionalized, the effect is variable, although primary amines consistently provide the highest potency. Conversion to secondary amines bearing small substituents (e.g., methyl, ethyl, cyclopropyl, cyclopropylmethyl, cyclobutylethyl, hydroxyethyl, *N,N*-dimethylaminoethyl, pyrrolidinylethyl, or acetyl) preserves activity but does not afford improvements. If the amine is separated from the aromatic ring by a methylene spacer or converted into a carboxamide, the activity is generally maintained. Larger or bulkier substituents containing terminal cyclic motifs are tolerated only when the terminal ring retains an NH group; for example, piperazinylethyl, piperazin-2-oxoethyl, and piperazine-carboxymethyl substituents maintain activity. In contrast, potency is markedly reduced with morpholinoethyl, morpholino-propyl, 3-fluoro-azetidinylethyl, 3,3-difluoro-azetidinylethyl, 3-methoxy-azetidinylethyl, 4-difluoro-piperidinylethyl, thiomorpholinylethyl, and thiomorpholine-sulfonyl derivatives, indicating pronounced steric and electronic constraints around this region. Finally, substitution on the heterobicyclic ring is well tolerated at the 4-position, where methyl, methoxy, cyano, and halogen groups maintain activity. In contrast, halogenation at the 6-position (Cl, Br, I) diminishes potency, and the presence of two halogens simultaneously at both 4- and 6-positions leads to a dramatic loss of activity, suggesting that steric encumbrance or unfavorable dipolar effects in this region impair productive binding. Biochemical inhibition of METTL1 catalytic activity was quantified using a RapidFire mass spectrometry–based assay measuring SAH production from SAM. In this context, compounds **3a**–**3g** displayed exceptional potency, with IC_50_ values ranging from 0.013 to 0.021 μM, placing them firmly within the low-nanomolar regime (Table 2).^146^ These data establish this subset as among the most potent METTL1/WDR4 inhibitors reported to date and confirm the THIQ-based scaffold as a privileged chemical architecture for targeting this enzyme complex. Cellular target engagement was further evaluated using the METTL1 InCellPulse assay in engineered HeLa cells expressing METTL1/WDR4 fused to an ePL tag. Compounds **3b**, **3c**, **3e**, and **3g** exhibited particularly strong intracellular engagement, with IC_50_ values between 4 and 7 nM, while **3a**, **3d**, and **3f** retained sub-20 nM potency (Table 2). The close concordance between biochemical and cellular IC_50_ values across this subset indicates that these molecules possess a favorable balance of membrane permeability, intracellular stability, and high intrinsic affinity for the native METTL1/WDR4 complex. Functional suppression of METTL1-mediated tRNA m^7^G modification was confirmed using the MSD m^7^G-46 assay in OE21 cells. In this physiologically integrated cellular system, compounds **3a**–**d** and **3g** demonstrated robust inhibition of m^7^G formation, with IC_50_ values below 0.05 μM, while **3e** and **3f** maintained sub-0.15 μM activity (Table 2). The consistent performance of these analogues across biochemical, target-engagement, and functional methylation assays underscores their ability to effectively inhibit METTL1 catalytic output in intact cellular environments. The biological consequences of METTL1 inhibition were further assessed in a 7 day CellTiter-Glo proliferation assay using OE21 esophageal squamous carcinoma cells, a model known to rely on METTL1-driven translational programs. All selected compounds inhibited cell proliferation at submicromolar concentrations. Notably, **3a**–**d** displayed the strongest antiproliferative effects, with IC_50_ values ranging from 0.123 to 0.395 μM, while **3e**–**g** also demonstrated clear growth–suppressive activity with IC_50_ values in the submicromolar range (Table 2). The overall alignment between enzymatic inhibition, cellular target engagement, functional m^7^G suppression, and antiproliferative efficacy strongly supports a causal relationship between METTL1/WDR4 inhibition and growth control in METTL1-dependent cancer cells. Collectively, compounds **3a**–**g** define a highly optimized subset of METTL1 inhibitors that combine nanomolar biochemical potency with robust intracellular activity and demonstrable phenotypic consequences. These findings represent the first clear demonstration that small-molecule inhibition of METTL1 can achieve sustained suppression of tRNA m^7^G modification and translate into antiproliferative effects in cancer cells, marking a significant milestone in the development of chemical probes and therapeutic leads targeting RNA epitranscriptomic regulation.^142^

A second series of compounds has been recently disclosed again by the company Storm Therapeutics in a patent demonstrating robust inhibitory activity against the METTL1–WDR4 RNA methyltransferase complex, as evidenced across enzymatic assays, target engagement studies, and cellular tests. These molecules consistently showed potent biochemical inhibition alongside measurable cellular efficacy, supported by direct target engagement through orthogonal assay platforms.^143^ From a structural standpoint, this chemotype is characterized by the same 1-methyl substituted THIQ core present in compounds **3a**–**g** bearing the same halogens (F, Cl) or electron-withdrawing groups such as CF_3_ at the 6- and/or 8-positions of the aromatic ring. Also in this series, the stereogenic center at C-1 can be present either as a racemic mixture or as isolated enantiomers (R or S), with the *S*-configuration generally showing a preference in terms of biological activity. This core is linked via an oxoethylamino spacer to the 6-position of a 1,2-dihydroisoquinolin-1-one moiety, forming a rigid yet tunable scaffold that appears well-suited for the engagement of the METTL1–WDR4 complex (Figure 9). Baseline activity is retained across this structural class. However, further optimization revealed that substitution at the 3-position of the dihydroisoquinolinone ring significantly enhances potency. In particular, the introduction of an aminomethyl group—either unsubstituted or bearing small alkyl substituents such as cyclopropylmethyl, cyclobutyl, or *tert*-butyl—results in notable activity gain. Additional improvements were observed upon incorporation of a fluorine atom at the 7-position of the dihydroisoquinolinone ring, suggesting a favorable electronic or conformational contribution to the binding affinity. The biological evaluation of this series reveals a coherent and informative activity profile across complementary assay formats, each probing a distinct aspect of compound performance. For clarity, the compounds that exhibited the most favorable profiles across all four assays are discussed here (compounds **4a–g,** Figure 9), and their corresponding IC_50_ values are summarized in Table 3.^143^ In biochemical assays measuring the inhibition of the METTL1–WDR4 catalytic activity, several compounds exhibited low nanomolar potency, with IC_50_ values in the 12–29 nM range (compounds **4a**–**g**). This level of activity indicates a highly efficient engagement of the enzymatic machinery and validates the core scaffold as a strong binder of the target complex. Importantly, this potency translates, albeit with some attenuation, into cellular target engagement as measured by thermal stabilization assays. Compounds such as **4c** and **4e** stand out, displaying sub-100 nM IC_50_ values (77 nM and 27 nM, respectively), indicative of efficient intracellular binding and favorable permeability or retention properties. Other analogues (e.g., **4a,b**, **4d** and **4f,g**) show good results even if they present a slight drop-off in this assay, suggesting that subtle structural differences may significantly impact cellular exposure or target accessibility despite comparable biochemical inhibitory potency. Functional inhibition of RNA methylation, assessed via reduction of m^7^G modification levels, generally occurs in the submicromolar range across the series. While this shift relative to biochemical potency is expected due to the increased complexity of the cellular environment, compounds such as **4c**, **4e** and **4g** maintain comparatively strong activity (IC_50_ ≈ 0.3–0.5 μM), supporting effective pathway modulation downstream of target binding.^143^ Finally, antiproliferative effects measured in OE21 cells reveal IC_50_ values generally spanning the mid- to high-submicromolar range (≈0.3–0.9 μM). Although these values are less potent than those observed in upstream assays, a consistent ranking is largely preserved. Compound **4c** emerges as particularly balanced, combining strong target engagement with one of the most favorable antiproliferative profiles (0.493 μM), while compound **4e**, despite superior in biochemical and engagement potency, shows slightly weaker translation into phenotypic effect (0.631 μM). This divergence may reflect differences in compound-specific pharmacokinetic properties at the intracellular level. Overall, the data set highlights a clear progression from high-affinity enzymatic inhibition to effective cellular target engagement and downstream functional activity, with compound **4e** representing the most potent inhibitor at the biochemical and target engagement level, and compound **4c** offering the most balanced overall profile across all assays. The observed discrepancies between assay formats provide valuable insight into structure–property relationships, particularly with respect to cellular permeability, target residence time, and functional efficacy, and will likely inform further optimization of this promising class of METTL1–WDR4 inhibitors.^143^

**Figure 9:**
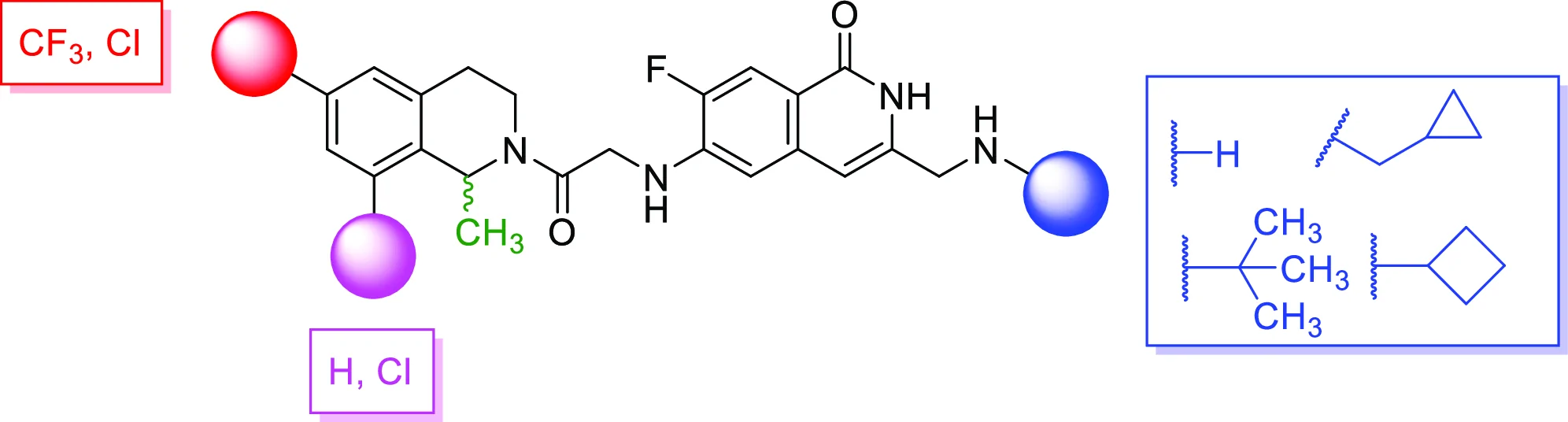
General structure for compounds **4a**–**g**.

**Table 3:**
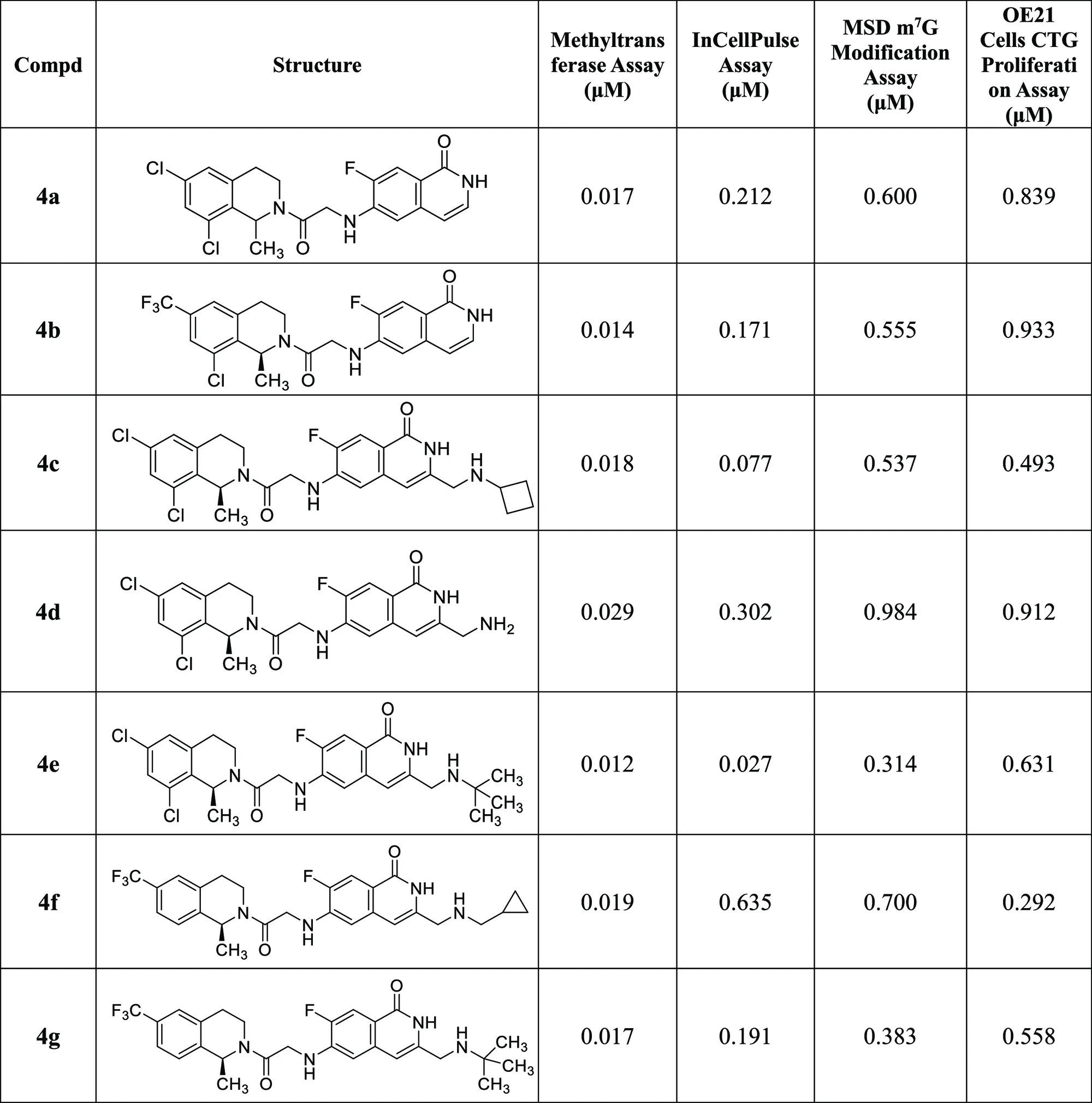
Structures and Biochemical/Biological Activity Profiles of Compounds **4a–g**

Despite the remarkable progress achieved in METTL1 inhibitor discovery, optimization of drug-like properties remains a critical prerequisite for clinical translation. The most advanced chemotypes reported to date, including tetrahydroisoquinoline (THIQ)-based and dihydroisoquinolinone derivatives, exhibit low-nanomolar inhibitory potency, robust cellular target engagement, and effective suppression of tRNA m^7^G methylation. However, publicly available information on their ADME/DMPK properties remains scarce, with comprehensive pharmacokinetic data not yet disclosed in either the primary literature or patent applications. From a medicinal chemistry perspective, future lead optimization should balance potency with physicochemical properties governing systemic exposure and tissue distribution. The polar functionalities required for SAM recognition may compromise passive permeability, whereas the relatively lipophilic fused bicyclic scaffolds could increase plasma protein binding and influence metabolic clearance. Consequently, early optimization should incorporate molecular weight, lipophilicity, ionization state, polar surface area, microsomal stability, CYP-mediated metabolism, and plasma protein binding alongside biochemical potency and selectivity. An additional consideration is tissue-specific drug delivery. Since METTL1 dysregulation has been implicated in aggressive gliomas, blood–brain barrier permeability may become an important design objective for selected therapeutic indications. Accordingly, future optimization strategies should integrate target potency, selectivity, and early ADME/DMPK profiling to maximize translational potential (Figure 10).

**Figure 10:**
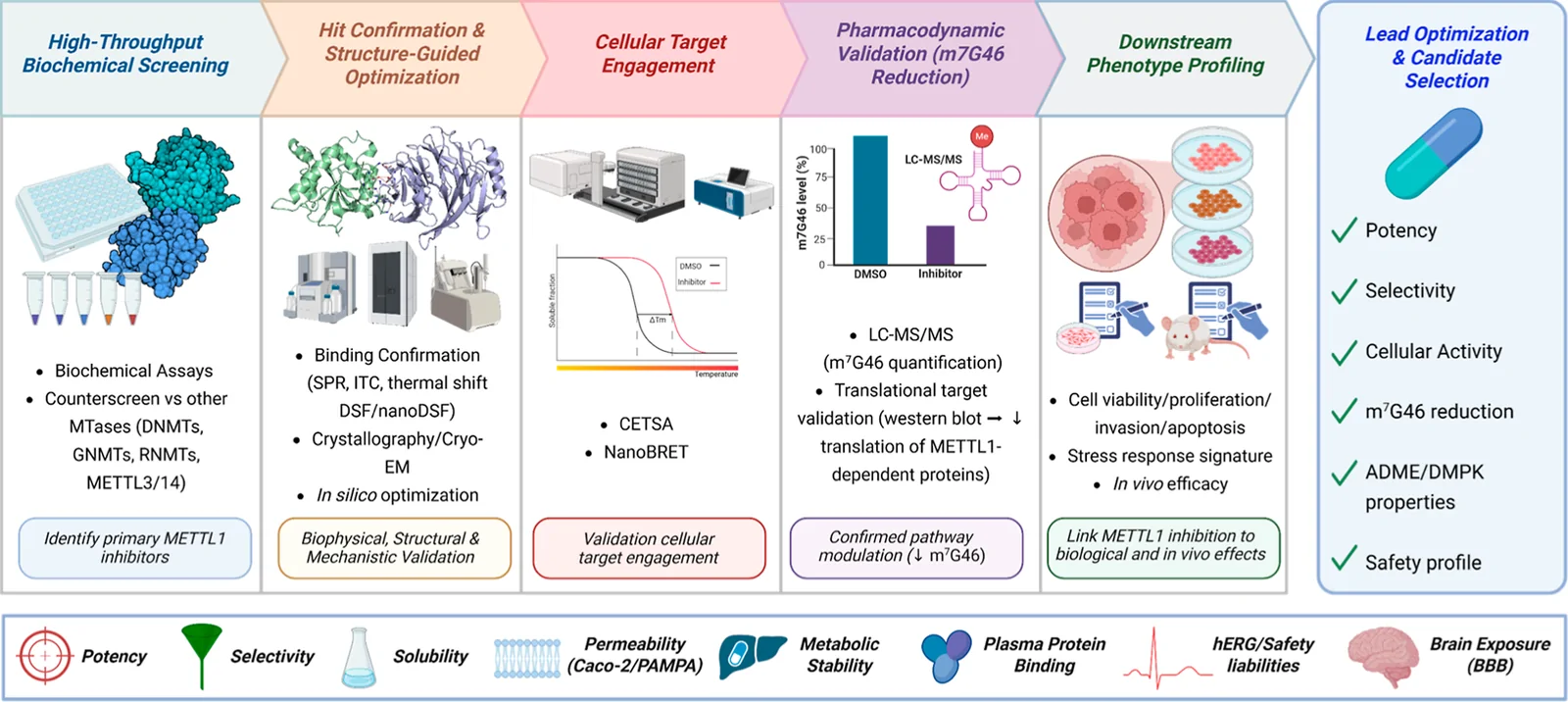
Proposed medicinal chemistry assay funnel for METTL1/WDR4 inhibitor discovery and optimization. The workflow integrates complementary biochemical, biophysical, cellular, pharmacodynamic, and phenotypic assays to support structure-guided lead optimization. Following primary biochemical screening and selectivity profiling, compounds are advanced through cellular target-engagement assays, validation of m^7^G46 reduction, and downstream functional studies before iterative optimization of potency, selectivity, ADME/DMPK properties, and safety to identify high-quality METTL1/WDR4 inhibitors. (Created in https://BioRender.com).

### Alternative Therapeutic Strategies for Indirect METTL1/WDR4 Targeting

Other therapeutic strategies are beginning to exploit the epitranscriptomic functions of METTL1 both directly and indirectly, opening new avenues for translational intervention.

Nanotherapeutic approaches represent a particularly innovative direction. A notable example is the PAE@5-FUts nanocomplex, which was designed to selectively deliver 5-fluorouracil (5-FU) to CRC cells with high METTL1 expression. In this setting, METTL1 promotes the generation of tsRNA-GlyGCC, a tRNA-derived fragment that contributes to 5-FU resistance. By preferentially targeting METTL1-high cells, the PAE@5-FUts system suppresses METTL1-induced tsRNA-GlyGCC and thereby restores chemosensitivity. Functionally, this nanocomplex lowers the IC_50_ of 5-FU from 28 μM to 6 μM and inhibits JAK1/STAT6 signaling in vivo, resulting in a robust antitumor effect.^144^ These data underscore that METTL1-dependent vulnerabilities can be exploited for precision nanomedicine. However, several hurdles must be overcome before such strategies can be translated clinically. Long-term safety and off-target toxicity require systematic evaluation, as prolonged interference with RNA methylation may have unpredictable effects in normal tissues. The heterogeneous architecture and perfusion of the tumor microenvironment also limit delivery efficiency and necessitate careful optimization of nanoparticle size, surface charge and formulation consistency. In addition, achieving tumor-selective uptake will likely depend on precise patient stratification and rational ligand engineering, such as decorating nanocarriers with aptamers or antibody-based targeting moieties. Future work in this area should prioritize smart, stimuli-responsive delivery platforms that respond to tumor-specific cues, as well as personalized design strategies that match nanocarrier characteristics to METTL1 expression levels and tumor biology.

Beyond direct inhibition, a complementary strategy involves targeting key pathways downstream of METTL1, where oncogenic outputs are more narrowly focused and may offer a broader therapeutic window. One major axis involves the EGFR pathway. In multiple METTL1-dependent tumors, METTL1 knockdown does not significantly alter EGFR mRNA abundance, but instead reduces EGFR translation efficiency by depleting m^7^G-dependent tRNA decoding capacity. As a result, EGFR protein levels are governed primarily at the translational level rather than by transcription or protein stability. Because EGFR protein half-life is not markedly affected, strategies that exclusively aim to accelerate EGFR degradation or block transcription are unlikely to fully counteract METTL1-driven oncogenic translation. Therapeutic approaches that blunt the translational efficiency of EGFR mRNAs, e.g., by interfering with codon-biased decoding or with METTL1-regulated tRNA pools, may offer a more rational and durable strategy to suppress EGFR signaling in METTL1-high cancers.^145,146^

A second key targetable axis is AKT signaling. m^7^G-modified tRNAs promote PI3K/AKT activation in a manner that is shaped by mutations in core upstream regulators such as EGFR and PTEN. Hyperactivated AKT, in turn, downregulates tumor-suppressive miRNAs containing m^7^G, including members of the let-7 family, thereby further accelerating tumor progression. EGFR autophosphorylation drives AKT activation, whereas restoration of PTEN attenuates METTL1-dependent oncogenic phenotypes, indicating a tight interdependence between METTL1, EGFR, PTEN, and PI3K/AKT. Pharmacologic inhibitors of PI3K, AKT, and CCNB1 have been shown to reduce AKT phosphorylation and impair EMT, especially in METTL1/WDR4-hyperactive contexts. These data suggest that optimal therapeutic regimens might combine METTL1 inhibition (or modulation of its downstream tRNA substrates) with agents targeting PI3K/AKT and compensating for EGFR/PTEN imbalances. Such dual targeting could simultaneously restrain AKT activation and dismantle the codon-biased translational program that sustains EMT and invasive behavior.^124,147^

The cell cycle machinery itself represents another critical downstream layer of METTL1/WDR4 signaling. METTL1 depletion consistently compromises the G_1_/S transition, whereas WDR4 loss disrupts G_2_/M progression, indicating that the METTL1/WDR4 complex coordinates multiple cell-cycle checkpoints. mRNAs encoding cyclins and CDKs are particularly enriched in AGA codons decoded by the METTL1-sensitive tRNA Arg-TCT-4–1. Proteomic profiling demonstrates preferential upregulation of AGA-rich proteins in METTL1/WDR4-hyperactive cells, linking tRNA m^7^G status directly to the abundance of cell-cycle regulators. Strikingly, overexpression of Arg-TCT-4–1 alone is sufficient to phenocopy METTL1/WDR4 hyperactivation and to drive malignant transformation, emphasizing that a single tRNA species can function as a potent oncogenic effector. This observation opens the possibility of developing therapeutic strategies that target tRNA Arg-TCT-4–1 itself, its biogenesis, or the decoding of AGA codons, thereby selectively dismantling METTL1-driven proliferative programs without completely abolishing global translation.^148,149^

Finally, specific miRNAs downstream of METTL1/WDR4 constitute attractive therapeutic candidates that integrate epitranscriptomic control with post-transcriptional gene regulation. METTL1 and WDR4 modulate miRNA biogenesis by installing m^7^G on pri-miRNAs such as let-7 family members and miR-149–3p, which enhances their maturation and alters their target spectrum. This layer of regulation is particularly intriguing because, although METTL1 overexpression is generally associated with poor prognosis, some METTL1-dependent miRNAs exert clear antitumor functions. For example, m^7^G-modified let-7 suppresses HMGA2, RAS and MYC, the three central oncogenic drivers across multiple tumor types. Similarly, in cisplatin-resistant CRC, METTL1-dependent maturation of miR-149–3p improves chemosensitivity by modulating the S100A4/p53 axis: miR-149–3p represses S100A4, stabilizes p53, and increases apoptosis in response to cisplatin. These miRNAs therefore represent mechanistically grounded therapeutic targets that lie downstream of METTL1. Therapeutic strategies could include miRNA mimics to restore tumor-suppressive METTL1-dependent miRNAs in contexts where their maturation is compromised, or combination regimens in which METTL1 modulation is coupled with delivery of specific miRNA mimics or inhibitors to fine-tune the balance between oncogenic and tumor-suppressive epitranscriptomic outputs.^11,150^

## Conclusion

The METTL1/WDR4 RNA methyltransferase complex occupies a unique position among RNA-modifying enzymes because it governs both tRNA structure and selective mRNA translation through m^7^G deposition. Its activity shapes the codon-biased proteomes and stress-response networks, making it an inherently attractive, yet mechanistically complex, therapeutic target for conditions such as cancer and neurodegenerative diseases where protein synthesis regulation is crucial.

From a translational and clinical perspective, METTL1 clearly emerges as an appealing node for drug, but moving it toward the clinic comes with important challenges. Tumors with high METTL1 expression, reported in settings such as bladder cancer and HCC, may be particularly dependent on METTL1 activity and thus most likely to benefit from direct inhibitors or METTL1-directed delivery platforms. At the same time, METTL1 sits at the intersection of core RNA metabolism and tissue homeostasis, meaning that “target attractiveness” and “target risk” are tightly coupled in this case.

Against this broad biological landscape, the emergence of the first *bona fide* METTL1 inhibitors is both promising and cautionary. Early work identified **SA91–0178 (2)** as a macrophage-active METTL1 inhibitor with clear in vivo anti-inflammatory and organ-protective effects, demonstrating that METTL1 can be pharmacologically modulated without overt toxicity. Parallel fragment-based and structure-guided efforts yielded minimalist adenine-derived scaffolds capable of engaging the SAM pocket with unexpectedly high ligand efficiency, confirming that METTL1 is chemically tractable despite the conserved architecture of class I methyltransferases.

The most compelling advance comes from the two recently disclosed THIQ-based series (WO2025/104443 and WO2025/248262), which establishes the first high-affinity, selective METTL1/WDR4 inhibitor classes. These compounds achieve nanomolar potency in biochemical and cellular target-engagement assays, suppress tRNA m^7^G formation in intact cells, and exhibit robust antiproliferative effects in METTL1-dependent ESCC models. Their consistent SARs, intracellular permeability, and functional activity provide a genuine chemical foothold for interrogating METTL1 biology and advancing translational studies. So, these developments confirm that METTL1 can indeed be targeted with small molecules, laying the groundwork for rigorous preclinical validation and the eventual design of therapeutic strategies that precisely modulate m^7^G-dependent epitranscriptomic circuits.

On the other hand, several limitations and open questions highlight why METTL1 must still be approached with caution. First, selectivity remains a major challenge. The SAM-binding site is conserved across many class I methyltransferases, and even the most advanced adenosine-like inhibitors show varying degrees of cross-reactivity with other methyltransferases. The two THIQ series appear more potent and likely selective, but a comprehensive off-target and chemoproteomic profile for both series is still lacking, while it is crucial for understanding their potential side effects and interactions with other methyltransferases. Second, the systemic consequences of chronic METTL1 inhibition are unknown. Given its roles in hematopoiesis, cardiac remodeling, neurodevelopment, immune regulation and stress responses, on-target toxicity in normal tissues is a realistic concern, particularly in rapidly proliferating or highly plastic compartments.

Another key limitation is biological rather than chemical: our understanding of whether METTL1 is a *bona fide* oncogenic addiction versus a context-specific guardian of genome stability remains incomplete. The same is true for its roles in cardiovascular and neurological disease, where it can be either pathogenic or protective depending on cell type and injury context. This duality argues strongly against indiscriminate systemic inhibition and instead favors carefully stratified, disease- and genotype-specific approaches. It also underscores the need for robust biomarkers, integrating METTL1/WDR4 expression, m^7^G-tRNA and mRNA signatures, codon-usage profiling and immune-context readouts, to identify patients most likely to benefit and to avoid those in whom METTL1 activity may be compensatory or protective.

Despite these caveats, there are several reasons why METTL1 remains a particularly compelling target to pursue. Mechanistically, it occupies an unusually privileged position at the interface of translation, RNA stability, miRNA biogenesis and stress signaling, allowing relatively modest changes in activity to propagate across multiple oncogenic and inflammatory circuits. Therapeutically, the fact that METTL1 primarily drives selective translational rewiring, rather than global shutdown of protein synthesis, suggests that partial inhibition may be sufficient to dismantle malignant programs while sparing most normal tissues. The success of early nanotherapeutic approaches and combination regimens, as well as the clear modulation of immune microenvironments upon METTL1 perturbation, further argues that METTL1 inhibitors could be rationally integrated with existing chemotherapies, targeted agents and immunotherapies to enhance efficacy and overcome resistance.

In this sense, METTL1 is more than “just another methyltransferase”: it is an epitranscriptomic hub whose drugging will illuminate fundamental principles of codon-biased translation and RNA-based signaling in human disease. The next phase of research should therefore couple high-quality chemical probes and advanced inhibitors with deep functional genomics, multiomics profiling and sophisticated in vivo models to map beneficial versus deleterious consequences of METTL1 inhibition/degradation across tissues. METTL1 has clear translational promise, but meaningful clinical implementation will require closing substantial knowledge gaps, especially around selectivity, immune context, and long-term on-target safety, through rigorous preclinical work and carefully designed clinical trials.

## Abbreviations Used

5-FU: 5-fluorouracil
7BS: seven-β-strand fold
ACC: adrenocortical carcinoma
AKI: acute kidney injury
AKT: Protein Kinase B
ALS: amyotrophic lateral sclerosis
AM: ameloblastoma
ANXA2: annexin A2
ASCL1: achaete-scute family BHLH transcription factor 1
ATF3: activating transcription factor 3
ATF5: activating transcription factor 5
BC: breast cancer
BCa: bladder cancer
BCAT1: branched-chain amino acid transaminase 1
BMDM: bone marrow–derived macrophage
CAD: coronary artery disease
CCNA2: cyclin A2
CCNB1: cyclin B1
CCND1: cyclin D1
CCND2: cyclin D2
CCND3: cyclin D3
CCNE1: cyclin E1
CD4^+^ T cell: CD4-positive T lymphocyte
CD8^+^ T cell: CD8-positive T lymphocyte
CDK1: cyclin-dependent kinase 1
CDK4/6: cyclin-dependent kinase 4/6
CDK6: cyclin-dependent kinase 6
CDK8: cyclin-dependent kinase 8
CEBPB: CCAAT enhancer binding protein beta
CHEK2: checkpoint kinase 2
CLP: cecal ligation and puncture
CRC: colorectal cancer
CRPC: castration-resistant prostate cancer
CTG: CellTiter-Glo
CTLA-4: cytotoxic T-lymphocyte–associated protein 4
CXCL5: C-X-C motif chemokine ligand 5
CXCL8: C-X-C motif chemokine ligand 8
CXCR2: C-X-C motif chemokine receptor 2
CYLD: cylindromatosis
CVDs: cardiovascular diseases
DIMT1L: DIMT1-like rRNA methyltransferase
DNA-PKcs: DNA-dependent protein kinase catalytic subunit
Drosha: ribonuclease III Drosha
E2F: E2F transcription factor
eEF1A: eukaryotic elongation factor 1A
EFEMP1: EGF-containing fibulin-like extracellular matrix protein 1
EIF2A: eukaryotic translation initiation factor 2A
eIF2α: eukaryotic translation initiation factor 2 alpha
eIF4E: eukaryotic translation initiation factor 4E
EIF4E2: eukaryotic translation initiation factor 4E family member 2
ERK1/2: extracellular signal-regulated kinases 1 and 2
ESCC: esophageal squamous cell carcinoma
EWG: electron-withdrawing group
F4/80^+^: F4/80-positive macrophages
FAM119B: family with sequence similarity 119 member B
FOXM1: forkhead box M1
FTH1: ferritin heavy chain 1
GADD45A: growth arrest and DNA damage-inducible alpha
GC: gastric cancer
GSK3β: glycogen synthase kinase 3 beta
HCC: hepatocellular carcinoma
HD: Huntington’s disease
HF: heart failure
HIF-1α: hypoxia-inducible factor 1 alpha
HMGA2: high mobility group AT-hook 2
HNSCC: head and neck squamous cell carcinoma
HK1: hexokinase 1
hiPSC: human induced pluripotent stem cell
ICC: intrahepatic cholangiocarcinoma
IFN: interferon
IL-1β: interleukin 1 beta
INCA1: inhibitor of CDK interacting with cyclin A1
I/R: ischemia–reperfusion
JAK1: Janus kinase 1
KIF5A: kinesin family member 5A
*K*_i_-67: marker of proliferation *K*_i_-67
LASSO: least absolute shrinkage and selection operator
LC: lung cancer
let-7: lethal-7 microRNA family
LIN28: LIN28 RNA-binding protein
lncRNA: long noncoding RNA
LOXL2: lysyl oxidase like 2
LSM14A: LSM14 homologue A
LS: liposarcoma
m^6^A: N^6^-methyladenosine
m^7^G: N^7^-methylguanosine
m^7^G46; METTL: methyltransferase-like
METTL1: methyltransferase-like 1
miRNA (miR): microRNA
miR-149–3p: microRNA 149–3p
MPD: microcephalic primordial dwarfism
MSC: multi–tRNA synthetase complex
mtiRL: m^7^G-modified tRNA-derived RNA Lys­(TTT)
mTORC1: mechanistic target of rapamycin complex 1
MYC (c-MYC): MYC proto-oncogene
MTDH: metadherin
NBL: neuroblastoma
NEK1: NIMA related kinase 1
NFATc4: nuclear factor of activated T cells cytoplasmic 4
NHEJ: nonhomologous end joining
NPC: nasopharyngeal carcinoma
OA: osteoarthritis
OC: ovarian cancer
OE21: human esophageal squamous cell carcinoma cell line
OS: osteosarcoma
OSCC: oral squamous cell carcinoma
OTX2: orthodenticle homeobox 2
OXPHOS: oxidative phosphorylation
PAE@5-FUts: poly­(β-amino ester)–5-fluorouracil–tsRNA nanocomplex
PAX6: paired box 6
PCa: prostate cancer
PD: Parkinson’s disease
PDCD10: programmed cell death 10
PD-L1: programmed death-ligand 1
PGK1: phosphoglycerate kinase 1
PKM: pyruvate kinase M
pri-miRNA: primary microRNA
PROTAC: proteolysis-targeting chimera
PTEN: phosphatase and tensin homologue
PTC: papillary thyroid carcinoma
PTPN13: protein tyrosine phosphatase nonreceptor type 13
PVT1: plasmacytoma variant translocation 1
RAW264.7: murine macrophage cell line
RB1: retinoblastoma protein 1
RFA: radiofrequency ablation
Ribo-seq: ribosome profiling sequencing
RNC-seq: ribosome nascent-chain sequencing
RNMT: RNA guanine-7 methyltransferase
RAM: RNMT-activating miniprotein
RPTOR: regulatory-associated protein of mTOR
RSK: ribosomal S6 kinase
SAH: *S*-adenosyl- *l* -homocysteine
SAM: *S*-adenosyl- *l* -methionine
SARM1: sterile alpha and TIR motif-containing protein 1
S100A4: S100 calcium binding protein A4
SOX1: SRY-box transcription factor 1
SP1: specificity protein 1
SPIB: Spi-B transcription factor
SPTBN2: spectrin beta nonerythrocytic 2
SRSF9: serine/arginine-rich splicing factor 9
STAT1: signal transducer and activator of transcription 1
STAT6: signal transducer and activator of transcription 6
TGF-β: transforming growth factor beta
THIQ: tetrahydroisoquinoline
tiRNA: tRNA-derived stress-induced RNA
TKI: tyrosine kinase inhibitor
TIME: tumor immune microenvironment
TLR7: Toll-like receptor 7
TMEM11: transmembrane protein 11
TRMT112: tRNA methyltransferase activator subunit 11–2
tsRNA: tRNA-derived small RNA
TROP2: trophoblast cell surface antigen 2
TSPAN31: tetraspanin 31
ULK1: Unc-51–like autophagy activating kinase 1
UTR: untranslated region
VEGFA: vascular endothelial growth factor A
WBSCR22: Williams–Beuren syndrome chromosome region 22
WDR4: WD repeat domain 4
WNT: wingless/integrated signaling pathway
Yes1: YES proto-oncogene 1.
